# An end to end workflow for differential gene expression using Affymetrix microarrays

**DOI:** 10.12688/f1000research.8967.2

**Published:** 2018-07-03

**Authors:** Bernd Klaus, Stefanie Reisenauer

**Affiliations:** 1 EMBL Heidelberg, Heidelberg, 69117, Germany

**Keywords:** microarray, gene expression

## Abstract

In this article, we walk through an end-to-end Affymetrix microarray differential expression workflow using Bioconductor packages. This workflow is directly applicable to current "Gene'' type arrays, e.g.the HuGene or MoGene arrays, but can easily be adapted to similar platforms. The data analyzed here is a typical clinical microarray data set that compares inflamed and non-inflamed colon tissue in two disease subtypes. For each disease, the differential gene expression between inflamed- and non-inflamed colon tissue was analyzed. We will start from the raw data CEL files, show how to import them into a Bioconductor ExpressionSet, perform quality control and normalization and finally differential gene expression (DE) analysis, followed by some enrichment analysis.

## Introduction

In this article we introduce a complete workflow for a typical (Affymetrix) microarray analysis. Data import, preprocessing, differential expression and enrichment analysis are discussed.

The data set used ^1^ is from a paper studying the differences in gene expression in inflamed and non-inflamed tissue. 14 patients suffering from Ulcerative colitis (UC) and 15 patients with Crohn’s disease (CD) were tested, and from each patient inflamed and non-inflamed colonic mucosa tissue was obtained via a biopsy. This is a typical clinical data set consisting of 58 arrays in total. Our aim is to analyze differential expression (DE) between the tissues. Our results show a substantial overlap with the results of the original paper.

## Workflow package installation

The workflow is wrapped in a package called maEndToEnd.

The maEndToEnd package can currently be obtained from GitHub and is available via the current development version of Bioconductor (3.8) (see here: http://bioconductor.org/packages/devel/workflows/html/maEndToEnd.html).

### Workflow package installation from Bioconductor

You can install the package via the biocLite function.

## try http:// if https:// URLs are not supported
source("https://bioconductor.org/biocLite.R")
biocLite("maEndToEnd")

Currently, the workflow is available in the development version of Bioconductor (3.8), which will become the release version in October 2018.

For details on how to use this version of Bioconductor see: http://bioconductor.org/developers/how-to/useDevel/

### Workflow package installation from Github

#In order to download the package from GitHub, we need the "install_github"
#function from the "remotes" package. We download the latest developer
#version of "remotes" from GitHub with the devtool::install_github
#function; note that this is necessary as the current "remotes" version on
#CRAN doesn’t allow us to correctly download the "maEndToEnd" package:

install.packages("devtools")
library(devtools)

devtools::install_github("r-lib/remotes")
library(remotes)
packageVersion("remotes") # has to be 1.1.1.9000 or later

remotes::install_github("b-klaus/maEndToEnd", ref="master")

### Workflow package import

Once the workflow package has been successfully installed, we can use a call to library() in order to load it. This will also load all the other packages neccessary to run the workflow.

suppressPackageStartupMessages({library("maEndToEnd")})

### List of packages required for the workflow

Below, you find a list of packages that are required by the workflow. Some Helper/Styling packages have been commented here, as they are not strictly neccesary to execute the workflow.

#General Bioconductor packages
     library(Biobase)
     library(oligoClasses)

#Annotation and data import packages
     library(ArrayExpress)
     library(pd.hugene.1.0.st.v1)
     library(hugene10sttranscriptcluster.db)

#Quality control and pre-processing packages
     library(oligo)
     library(arrayQualityMetrics)

#Analysis and statistics packages
     library(limma)
     library(topGO)
     library(ReactomePA)
     library(clusterProfiler)

#Plotting and color options packages
     library(gplots)
     library(ggplot2)
     library(geneplotter)
     library(RColorBrewer)
     library(pheatmap)

#Formatting/documentation packages
   #library(rmarkdown)
   #library(BiocStyle)
    library(dplyr)
    library(tidyr)

#Helpers:
     library(stringr)
     library(matrixStats)
     library(genefilter)
     library(openxlsx)
    #library(devtools)

## Downloading the raw data from ArrayExpress

The first step of the analysis is to download the raw data CEL files. These files are produced by the array scanner software and contain the measured probe intensities. The data we use have been deposited at ArrayExpress and have the accession code **E-MTAB-2967**.

We will store these files in the directory **raw_data_dir** which defaults to a temporary directory.

raw_data_dir <- tempdir()

if (!dir.exists(raw_data_dir)) {
    dir.create(raw_data_dir)
}

Each ArrayExpress data set has a landing page summarizing the data set, and we use the getAEfunction from the *ArrayExpress* Bioconductor package to obtain the ftp links to the raw data files ( Data from Palmieri et. al. on ArrayEpress).

With the code below, we download the raw data (also including annotation data) from ArrayExpress ^2^ by using the getAE-function. The data are saved in the raw_data_dir created above. The names of the downloaded files are returned as a list.

anno_AE <- getAE("E-MTAB-2967", path = raw_data_dir, type = "raw")

We will now have a closer look at the data we downloaded from ArrayExpress

## Background information on the data

### Information stored in ArrayExpress

Each dataset at ArrayExpress is stored according to the MAGE-TAB (MicroArray Gene Expression Tabular) specifications as a collection of tables bundled with the raw data. The MAGE-TAB format specifies up to five different types of files:

- **Investigation Description Format (IDF)**
- **Array Design Format (ADF)**
- **Sample and Data Relationship Format (SDRF)**
- **raw data files**
- **processed data files**

Other than the raw data files, the IDF and the SDRF file are important for us. The IDF file contains top level information about the experiment including title, description, submitter contact details and protocols. The SDRF file contains essential information on the experimental samples, e.g. the experimental group(s) they belong to.

Before we move on to the actual raw data import, we will briefly introduce the ExpressionSet class contained in the *Biobase* package. It is commonly used to store microarray data in Bioconductor.

### Bioconductor ExpressionSets

Genomic data can be very complex, usually consisting of a number of different components, e.g. information on the experimental samples, annotation of genomic features measured as well as the experimental data itself. In Bioconductor, the approach is taken that these components should be stored in a single structure to easily manage the data.

The package *Biobase* contains standardized data structures to represent genomic data. The ExpressionSet class is designed to combine several different sources of information (i.e. as contained in the various MAGE-TAB files) into a single convenient structure. An ExpressionSet can be manipulated (e.g., subsetted, copied), and is the input to or output of many Bioconductor functions.

The data in an ExpressionSet consist of:

- **assayData**: Expression data from microarray experiments with microarray probes in rows and sample identifiers in columns
- **metaData**
  – **phenoData**: A description of the samples in the experiment with sample identifiers in rows and description elements in columns; holds the content of the SDRF file
  – **featureData**: metadata about the features on the chip or technology used for the experiment with same rows as assayData by default and freely assignable columns
  – further annotations for the features, for example gene annotations from biomedical databases (annotation).
- **experimentData**: A flexible structure to describe the experiment.

**Figure 1.**
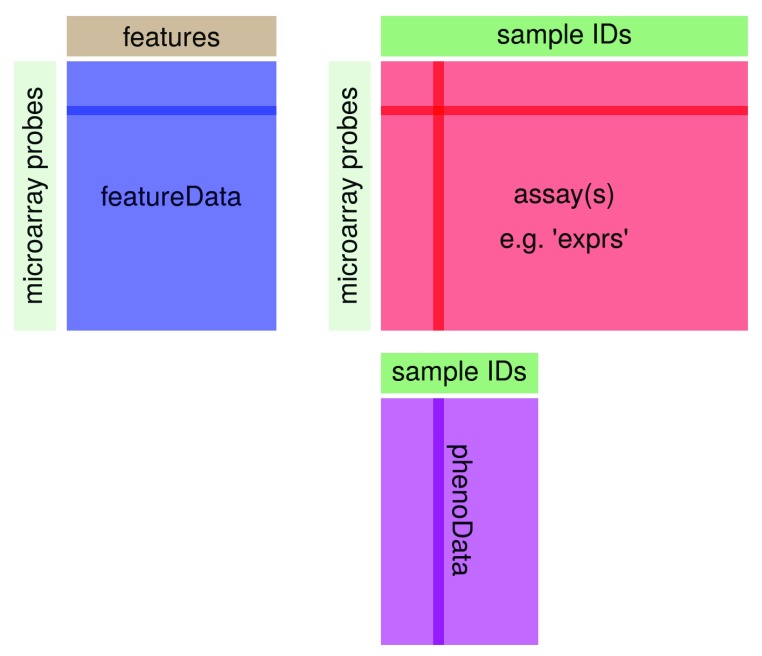
Structure of Bioconductor’s ExpressionSet class.

The ExpressionSet class coordinates all of these data, so that one does not have to worry about the details. However, one should keep in mind that the rownames of the phenoData have to match the column names of the assay data, while the row names of the assay data have to match the row names of the featureData. This is illustrated in Figure 1.

You can use the functions pData and fData to extract the sample and feature annotation, respectively, from an ExpressionSet. The function exprs will return the expression data itself as a matrix.

## Import of annotation data and microarray expression data as “ExpressionSet”

We import the SDRF file with the read.delim function from the raw data folder in order to obtain the sample annotation.

The sample names are given in the column Array.Data.File of the SDRF data table and will be used as rownames for the SDRF file.

We turn the SDRF table into an AnnotatedDataFrame from the *Biobase* package that we will need later to create an ExpressionSet for our data ^3^.

sdrf_location <- file.path(raw_data_dir, "E-MTAB-2967.sdrf.txt")
SDRF <- read.delim(sdrf_location)

rownames(SDRF) <- SDRF$Array.Data.File
SDRF <- AnnotatedDataFrame(SDRF)

We now create the Expression Set object raw_data, which contains array data, pheno data (from the SDRF file) as well as the information of the chip annotation package used.

The analysis of Affymetrix arrays starts with CEL files. These are the result of the processing of the raw image files using the Affymetrix software and contain estimated probe intensity values. Each CEL file additionally contains some metadata, such as a chip identifier.

We use the function read.celfiles from the *oligo* package ^4^ to import the files:

raw_data <- oligo::read.celfiles(filenames = file.path(raw_data_dir,
                                                      SDRF$Array.Data.File),
                                         verbose = FALSE, phenoData = SDRF)
stopifnot(validObject(raw_data))

This automatically creates an ExpressionSet, fills the sections “array data” with the data from the CEL files and uses the correct chip annotation package, in this case *pd.hugene.1.0.st.v1* (the chip-type is also stored in the .CEL files).

Furthermore, we specified our AnnotatedDataFrame“ SDRF” created earlier from the SDRF file as phenoData. Thus, we had to make sure to import the CEL files in the order that corresponds to the SDRF table — to enforce this, we used the column Array.Data.File of the SDRF table as the filenames argument.

Finally, we checked whether the object created is valid (e.g. sample names match between the different tables).

We now have a first look on the raw data.

The pData function of the Biobase package directly accesses the phenoData in the ExpressionSet raw_data. With the head() function, we can view the first six lines of the table. We have a look at the columns included and retain only those columns that are related to the experimental factors of interest.

head(Biobase::pData(raw_data))

               Source.Name Characteristics.individual. Characteristics.organism.
   164_I_.CEL	     164_I	                   164	            Homo sapiens
   164_II.CEL       164_II	                   164	            Homo sapiens
   183_I.CEL         183_I	                   183	            Homo sapiens
   183_II.CEL       183_II	                   183	            Homo sapiens
   2114_I.CEL       2114_I	                  2114	            Homo sapiens
   2114_II.CEL	   2114_II	                  2114	            Homo sapiens
               Characteristics.disease. Characteristics.organism.part.
   164_I_.CEL           Crohn’s disease                          colon
   164_II.CEL           Crohn’s disease                          colon
   183_I.CEL            Crohn’s disease                          colon
   183_II.CEL           Crohn’s disease                          colon
   2114_I.CEL           Crohn’s disease                          colon
   2114_II.CEL          Crohn’s disease	                         colon
                Characteristics.phenotype. Material.Type Protocol.REF
   164_I_.CEL  non-inflamed colonic mucosa organism part P-MTAB-41361
   164_II.CEL      inflamed colonic mucosa organism part P-MTAB-41361
   183_I.CEL   non-inflamed colonic mucosa organism part P-MTAB-41361
   183_II.CEL      inflamed colonic mucosa organism part P-MTAB-41361
   2114_I.CEL  non-inflamed colonic mucosa organism part P-MTAB-41361
   2114_II.CEL     inflamed colonic mucosa organism part P-MTAB-41361
               Protocol.REF.1 Extract.Name Protocol.REF.2 Labeled.Extract.Name
   164_I_.CEL    P-MTAB-41363	     164_I   P-MTAB-41364	  164_I:Biotin
   164_II.CEL    P-MTAB-41363	    164_II   P-MTAB-41364        164_II:Biotin
   183_I.CEL     P-MTAB-41363	     183_I   P-MTAB-41364         183_I:Biotin
   183_II.CEL    P-MTAB-41363	    183_II   P-MTAB-41364        183_II:Biotin
   2114_I.CEL    P-MTAB-41363	    2114_I   P-MTAB-41364        2114_I:Biotin
   2114_II.CEL   P-MTAB-41363	   2114_II   P-MTAB-41364       2114_II:Biotin
                 Label Protocol.REF.3 Assay.Name Technology.Type Array.Design.REF
   164_I_.CEL  biotin    P-MTAB-41366     164_I_     array assay       A-AFFY-141
   164_II.CEL  biotin    P-MTAB-41366     164_II     array assay       A-AFFY-141
   183_I.CEL   biotin    P-MTAB-41366      183_I     array assay       A-AFFY-141
   183_II.CEL  biotin    P-MTAB-41366     183_II     array assay       A-AFFY-141
   2114_I.CEL  biotin    P-MTAB-41366     2114_I     array assay       A-AFFY-141
   2114_II.CEL biotin    P-MTAB-41366    2114_II     array assay       A-AFFY-141
   	       Term.Source.REF Protocol.REF.4 Array.Data.File
   164_I_.CEL     ArrayExpress   P-MTAB-41367      164_I_.CEL
   164_II.CEL     ArrayExpress   P-MTAB-41367      164_II.CEL
   183_I.CEL      ArrayExpress   P-MTAB-41367       183_I.CEL
   183_II.CEL     ArrayExpress   P-MTAB-41367      183_II.CEL
   2114_I.CEL     ArrayExpress   P-MTAB-41367      2114_I.CEL
   2114_II.CEL    ArrayExpress   P-MTAB-41367     2114_II.CEL
                                                                                   Comment..ArrayExpress.FTP.file.
   164_I_.CEL  ftp://ftp.ebi.ac.uk/pub/databases/microarray/data/experiment/MTAB/E-MTAB-2967/E-MTAB-2967.raw.1.zip
   164_II.CEL  ftp://ftp.ebi.ac.uk/pub/databases/microarray/data/experiment/MTAB/E-MTAB-2967/E-MTAB-2967.raw.1.zip
   183_I.CEL   ftp://ftp.ebi.ac.uk/pub/databases/microarray/data/experiment/MTAB/E-MTAB-2967/E-MTAB-2967.raw.1.zip
   183_II.CEL  ftp://ftp.ebi.ac.uk/pub/databases/microarray/data/experiment/MTAB/E-MTAB-2967/E-MTAB-2967.raw.1.zip
   2114_I.CEL  ftp://ftp.ebi.ac.uk/pub/databases/microarray/data/experiment/MTAB/E-MTAB-2967/E-MTAB-2967.raw.1.zip
   2114_II.CEL ftp://ftp.ebi.ac.uk/pub/databases/microarray/data/experiment/MTAB/E-MTAB-2967/E-MTAB-2967.raw.1.zip
               Factor.Value.disease.     Factor.Value.phenotype. 
   164_I_.CEL        Crohn’s disease non-inflamed colonic mucosa 
   164_II.CEL        Crohn’s disease     inflamed colonic mucosa 
   183_I.CEL         Crohn’s disease non-inflamed colonic mucosa 
   183_II.CEL        Crohn’s disease     inflamed colonic mucosa
   2114_I.CEL        Crohn’s disease non-inflamed colonic mucosa
   2114_II.CEL       Crohn’s disease     inflamed colonic mucosa

The columns of interest for us are the following:

identifiers of the individuals, i.e. columns “Source.Name”, “Characteristics.individual.”
disease of the individual, i.e. “Factor.Value.disease.”
mucosa type, i.e. “Factor.Value.phenotype.”

We now subselect the corresponding columns:

Biobase::pData(raw_data) <- Biobase::pData(raw_data)[, c("Source.Name",
                                          "Characteristics.individual.",
                                          "Factor.Value.disease.",
                                          "Factor.Value.phenotype.")]



## Quality control of the raw data

The first step after the initial data import is the quality control of the data. Here we check for outliers and try to see whether the data clusters as expected, e.g. by the experimental conditions. The expression intensity values are in the assayData sub-object “exprs” and can be accessed by the exprs(raw_data) function. The rows represent the microarray probes, i.e. the single DNA locations on the chip, while the columns represent one microarray, i.e. a sample of inflamed and non-inflamed tissue of every patient, respectively.

Biobase::exprs(raw_data)[1:5, 1:5]

     164_I_.CEL 164_II.CEL 183_I.CEL 183_II.CEL 2114_I.CEL
   1       4496       5310      4492       4511       2872
   2        181        280       137        101         91
   3       4556       5104      4379       4608       2972
   4        167        217        99         79         82
   5         89        110        69         58         47

For quality control, we take the log2 of Biobase::exprs(raw_data), as expression data is commonly analyzed on a logarithmic scale.

**Figure 2.**
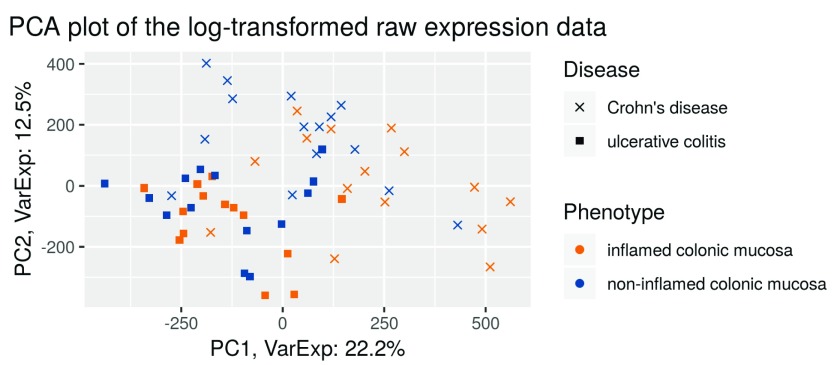
PCA plot of the log–transformed raw expression data.

We then perform a principal component analysis (PCA) and plot it ( Figure 2). Every point in the plot represents one sample, with the colour indicating the mucosa type (inflamed vs non-inflamed) and the shape indicating the disease (UC or CD).

exp_raw <- log2(Biobase::exprs(raw_data))
PCA_raw <- prcomp(t(exp_raw), scale. = FALSE)

percentVar <- round(100*PCA_raw$sdev^2/sum(PCA_raw$sdev^2),1)
sd_ratio <- sqrt(percentVar[2] / percentVar[1])

dataGG <- data.frame(PC1 = PCA_raw$x[,1], PC2 = PCA_raw$x[,2],
                       Disease = pData(raw_data)$Factor.Value.disease.,
                       Phenotype = pData(raw_data)$Factor.Value.phenotype.,
                       Individual = pData(raw_data)$Characteristics.individual.)

ggplot(dataGG, aes(PC1, PC2)) +
       geom_point(aes(shape = Disease, colour = Phenotype)) +
  ggtitle("PCA plot of the log-transformed raw expression data") +
  xlab(paste0("PC1, VarExp: ", percentVar[1], "%")) +
  ylab(paste0("PC2, VarExp: ", percentVar[2], "%")) +
  theme(plot.title = element_text(hjust = 0.5))+
  coord_fixed(ratio = sd_ratio) +
  scale_shape_manual(values = c(4,15)) +
  scale_color_manual(values = c("darkorange2", "dodgerblue4"))

The PCA plot ( Figure 2, performed on the log-intensity scale) of the raw data shows that the first principal component differentiates between the diseases. This means that the disease type is a major driver of gene expression differences. This might hinder our analysis, as we want to analyze the differential expression between inflamed and non-inflamed tissues, independently of the disease a person suffers from.

**Figure 3.**
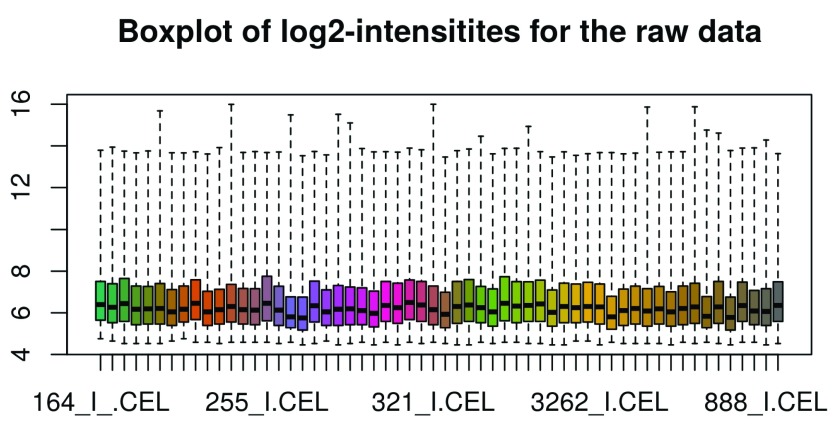
Intensity boxplots of the log2–transformed raw data.

We also represent the probe intensities via a boxplot graph with one box per individual microarray. ( Figure 3). Note that the oligo::boxplot function, i.e. the boxplot function of the oligo package, can take expression sets as argument. It accesses the expression data and performs a log2-transformation by default. We therefore can use raw_data as argument here.

oligo::boxplot(raw_data, target = "core",
                 main = "Boxplot of log2-intensitites for the raw data")

When looking at the boxplot ( Figure 3), we see that the intensity distributions of the individual arrays are quite different, indicating the need for an appropriate normalization, which we will discuss next.

Until now, we have only performed a very basic quality control; more elaborate quality control plots are available in the package *arrayQualityMetrics* ^5^. The package produces an html report, containing the quality control plots together with a description of their aims and an identification of possible outliers. We do not discuss this tool in detail here, but simply provide the code below, which creates a report for our raw data.

arrayQualityMetrics(expressionset = raw_data,
     outdir = tempdir(),
     force = TRUE, do.logtransform = TRUE,
     intgroup = c("Factor.Value.disease.", "Factor.Value.phenotype."))

## Background adjustment, calibration, summarization and annotation

### Background adjustment

After the initial import and quality assessment, the next step in processing of microarray data is background adjustment. This is essential because a proportion of the measured probe intensities are due to non-specific hybridization and the noise in the optical detection system. Therefore, observed intensities need to be adjusted to give accurate measurements of specific hybridization.

### Across-array normalization (calibration)

Normalization across arrays is needed in order to be able to compare measurements from different array hybridizations due to many obscuring sources of variation. These include different efficiencies of reverse transcription, labeling or hybridization reactions, physical problems with the arrays, reagent batch effects, and laboratory conditions.

### Summarization

After normalization, summarization is necessary to be done because on the Affymetrix platform, transcripts are represented by multiple probes, that is multiple locations on the array. For each gene, the background-adjusted and normalized intensities of all probes need to be summarized into one quantity that estimates an amount proportional to the amount of RNA transcript.

After the summarization step, the summarized data can be annotated with various information, e.g. gene symbols and ENSEMBL gene identifiers. There is an annotation database available from Bioconductor for our platform, namely the package *hugene10sttranscriptcluster.db*.

You can view its content like this:

head(ls("package:hugene10sttranscriptcluster.db"))

   [1] "hugene10sttranscriptcluster"
   [2] "hugene10sttranscriptclusterACCNUM"
   [3] "hugene10sttranscriptclusterALIAS2PROBE"
   [4] "hugene10sttranscriptclusterCHR"
   [5] "hugene10sttranscriptclusterCHRLENGTHS"
   [6] "hugene10sttranscriptclusterCHRLOC"

Additional information is available from the reference manual of the package. Essentially, the package provides a mapping from the transcript cluster identifiers to the various annotation data.

### Old and new “probesets” of Affymetrix microarrays

Traditionally, Affymetrix arrays (the so-called 3’ IVT arrays) were probeset based: a certain fixed group of probes were part of a probeset which represented a certain gene or transcript (note however, that a gene can be represented by multiple probesets).

The more recent “Gene” and “Exon” Affymetrix arrays are exon based and hence there are two levels of summarization to get to the gene level. The “probeset” summarization leads to the exon level. The gene/transcript level is given by “transcript clusters”. Hence, the appropriate annotation package for our chip type is called *hugene10sttranscriptcluster.db*.

**Figure 4.**
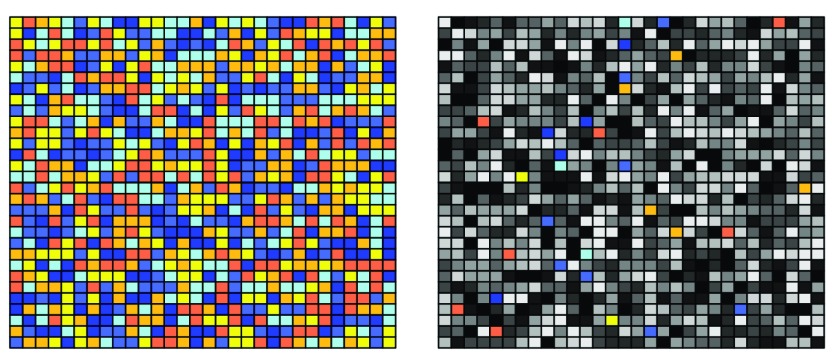
Visualization of the difference between "Exon" type array (left) and "Gene" type array (right).

“Gene” arrays were created as affordable versions of the “Exon” arrays, by only taking the “good” probes from the Exon array. Initially on the Exon array, at least four probes were part of one “Exon”. With the thinned out “Gene” array, many probesets were made up of three or fewer probes. This is visualized in Figure 4: Single probesets are indicated by single colours; probesets representing one gene are indicated by a colour shade: e.g., all yellow probes belong to one Exon, and all yellow, orange and red probesets belong to one gene:

On the left side, we see plenty of probes for each Exon/probeset (i.e. each colour): therefore, a summarization on the probeset/exon level makes sense. In the gene type array, however, only a small proportion of the original probes per probeset is included. Thus, a summarization on the probeset/exon level is not recommended for “Gene” arrays but nonetheless possible by using the *hugene10stprobeset.db* annotation package.

Note that furthermore, there are also no longer designated match/mismatch probes present on “Gene” and “Exon” type chips. The mismatch probe was initially intended as base-level for background correction, but hasn’t prevailed due to more elaborate background correction techniques that do not require a mismatch probe.

### One-step preprocessing in oligo

The package *oligo* allows us to perform background correction, normalization and summarization in one single step using a deconvolution method for background correction, quantile normalization and the RMA (robust multichip average) algorithm for summarization.

This series of steps as a whole is commonly referred to as RMA algorithm, although strictly speaking RMA is merely a summarization method ^6– 8^.

## Relative Log Expression data quality analysis

Before calibrating and evaluating the data, we want to perform another quality control procedure, namely Relative Log Expression (RLE), as described in the article by Gandolfo *et al*. ^9^. To this end, we first perform an RMA without prior normalization:

palmieri_eset <- oligo::rma(raw_data, target = "core", normalize = FALSE)

   Background correcting
   Calculating Expression

Further details on the RMA algorithm will be provided after RLE analysis, when the “full” RMA is carried out, including normalization.

The RLE is performed by calculating the median log2 intensity of every transcript across all arrays.

We do this by calculating the row medians of exprs(palmieri_eset), as the transcripts are represented by the rows and the single microarrays by the columns.

Note that we do not have to apply the log2 manually, as the output data of the RMA function is in log2 scale by default.

We then substract this transcript median intensity from every transcript intensity via the sweep function.

We then reshape the data into a format in which we can use to create a boxplot for each array, as before:

row_medians_assayData <- 
  Biobase::rowMedians(as.matrix(Biobase::exprs(palmieri_eset)))

RLE_data <- sweep(Biobase::exprs(palmieri_eset), 1, row_medians_assayData)

RLE_data <- as.data.frame(RLE_data)

RLE_data_gathered <-
  tidyr::gather(RLE_data, patient_array, log2_expression_deviation)

ggplot2::ggplot(RLE_data_gathered, aes(patient_array,
                                            log2_expression_deviation)) + 
  geom_boxplot(outlier.shape = NA) + 
  ylim(c(-2, 2)) + 
  theme(axis.text.x = element_text(colour = "aquamarine4",
                                      angle = 60, size = 6.5, hjust = 1 ,
                                      face = "bold"))


**Figure 5.**
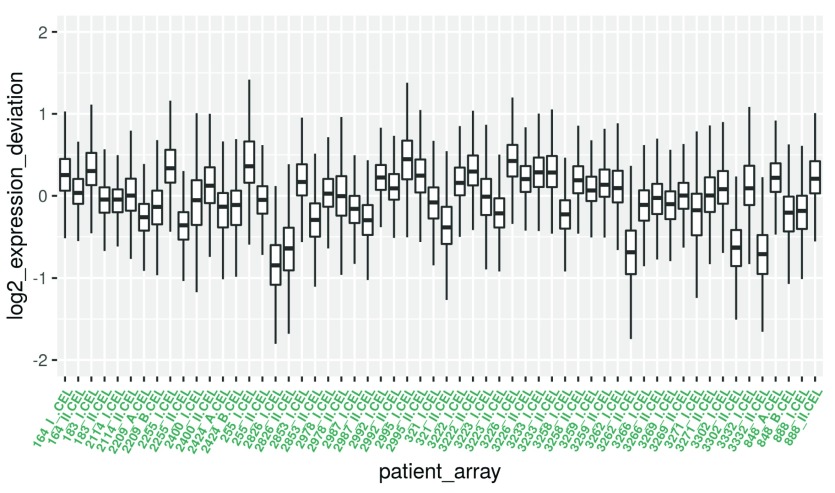
Boxplot for the RLE values.

Note that the y-axis now displays for each microarray the deviation of expression intensity from the median expression of the respective single transcripts across arrays.

Boxes with a larger extension therefore indicate an unusually high deviation from the median in a lot of transcripts, suggesting that these arrays are different from most of the others in some way.

Boxes that are shifted in y-direction indicate a systematically higher or lower expression of the majority of transcripts in comparison to most of the other arrays. This could be caused by quality issues or batch effects.

Therefore, if shape and median of a given box varies too much from the bulk, they should be inspected and potentially removed.

By inspecting the boxplot in Figure 5, five arrays could be considered as outliers: 2826_I, 2826_II, 3262_II, 3302_II and 3332_II are negatively y-shifted.

We will keep these samples in mind for heatmap cluster analysis later on in the workflow. Arrays that are confirmed to be outliers by heatmap analysis could be removed for subsequent analysis.

## RMA calibration of the data

Now, we can apply the full RMA algorithm to our data in order to background-correct, normalize and summarize:

palmieri_eset_norm <- oligo::rma(raw_data, target = "core")

   Background correcting
   Normalizing
   Calculating Expression

The parameter target defines the degree of summarization, the default option of which is “core”, using transcript clusters containing “safely” annotated genes. Other options for target include “extended” and “full”. For summarization on the exon level (not recommended for Gene arrays), one can use “probeset” as the target option. Although other methods for background correction and normalization exist, RMA is usually a good default choice. RMA shares information across arrays and uses the versatile quantile normalization method that will make the array intensity distributions match. However, it is preferable to apply it only after outliers have been removed. The quantile normalization algorithm used by RMA works by replacing values by the average of identically ranked (within a single chip) values across arrays. A more detailed description can be found on the Wikipedia page.

An alternative to quantile normalization would be the *vsn* algorithm,that performs background correction and normalization by robustly shifting and scaling intensity values within arrays before log-transforming them. This is less “severe” than quantile normalization ^10^.

### Some mathematical background on normalization (calibration) and background correction

A generic model for the value of the intensity *Y* of a single probe on a microarray is given by

*Y* = *B* + *α · S*

where B is a random quantity due to background noise, usually composed of optical effects and non-specific binding, *α* is a gain factor, and *S* is the amount of measured specific binding. The signal *S* is considered a random variable as well and accounts for measurement error and probe effects. The measurement error is typically assumed to be multiplicative so we can write:

                                                                        log( *S*) = *θ* + *φ* + *ε*

Here *θ* represents the logarithm of the true abundance, *φ* is a probe-specific effect, and *ε* accounts for the nonspecific error. This is the additive-multiplicative-error model for microarray data used by RMA and also the *vsn* algorithm ^10^. The algorithms differ in the way that *B* is removed and an estimate of *θ* is obtained.

### Quality assessment of the calibrated data

We now produce a clustering heatmap and another PCA plot using the calibrated data.

***PCA analysis.*** First, we perform a PCA analysis of the calibrated data analogously to the one with the raw data:

exp_palmieri <- Biobase::exprs(palmieri_eset_norm)
PCA <- prcomp(t(exp_palmieri), scale = FALSE)

percentVar <- round(100*PCA$sdev^2/sum(PCA$sdev^2),1)
sd_ratio <- sqrt(percentVar[2] / percentVar[1])

dataGG <- data.frame(PC1 = PCA$x[,1], PC2 = PCA$x[,2],
                      Disease =
                       Biobase::pData(palmieri_eset_norm)$Factor.Value.disease.,
                      Phenotype =
                       Biobase::pData(palmieri_eset_norm)$Factor.Value.phenotype.)


ggplot(dataGG, aes(PC1, PC2)) +
       geom_point(aes(shape = Disease, colour = Phenotype)) +
  ggtitle("PCA plot of the calibrated, summarized data") +
  xlab(paste0("PC1, VarExp: ", percentVar[1], "%")) +
  ylab(paste0("PC2, VarExp: ", percentVar[2], "%")) +
  theme(plot.title = element_text(hjust = 0.5)) +
  coord_fixed(ratio = sd_ratio) +
  scale_shape_manual(values = c(4,15)) +
  scale_color_manual(values = c("darkorange2", "dodgerblue4"))

**Figure 6.**
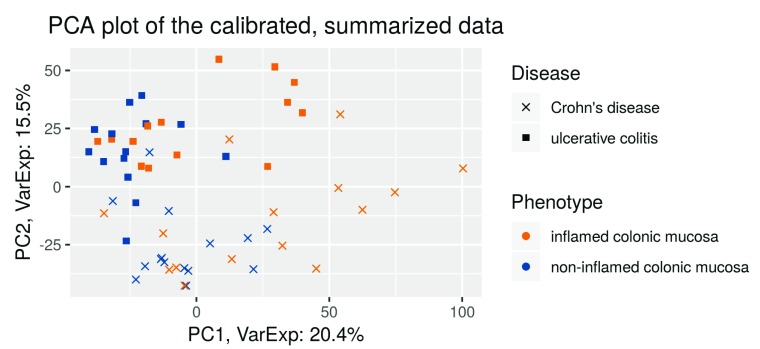
PCA plot of the calibrated, summarized data.

In comparison to the first PCA analysis before RMA ( Figure 2), we see that now the first principal component separates between the tissues types ( Figure 6). This indicates that now differential expression between the tissue types is the dominant source of variation. Note that the second principal component separates the diseases.

***Heatmap clustering analysis.*** We want to plot a heatmap with the sample-to-sample distances with the sample names as row-names. Also, we want to see how well the samples cluster for phenotype (inflamed and non-inflamed tissue) and disease (UC and CD), respectively. We use row annotation for that: it means that these features get a colour code and will be displayed to the left of each row.

phenotype_names <- ifelse(str_detect(pData
                                        (palmieri_eset_norm)$Factor.Value.phenotype.,
                                 "non"), "non_infl.", "infl.")
                                    
disease_names <- ifelse(str_detect(pData
                                        (palmieri_eset_norm)$Factor.Value.disease.,
                                "Crohn"), "CD", "UC")

annotation_for_heatmap <-
   data.frame(Phenotype = phenotype_names, Disease = disease_names)
   
row.names(annotation_for_heatmap) <- row.names(pData(palmieri_eset_norm))

In order to map the sample-to-sample distances, we first compute the distances using the dist function. We need to transpose the expression values since the function computes the distances between the rows (i.e. genes in our case) by default. The default distance is the Euclidean one. However this can be changed and we choose the Manhattan distance here (it uses absolute distances along rectangular paths instead of squared distances of the direct path), as it is more robust. We set the diagonal of the distance matrix to NA in order to increase the contrast of the color coding. Those diagonal entries do not contain information since the distance of a sample to itself is always equal to zero.

dists <- as.matrix(dist(t(exp_palmieri), method = "manhattan"))

rownames(dists) <- row.names(pData(palmieri_eset_norm))
hmcol <- rev(colorRampPalette(RColorBrewer::brewer.pal(9, "YlOrRd"))(255))
colnames(dists) <- NULL
diag(dists) <- NA

ann_colors <- list(
  Phenotype = c(non_infl. = "chartreuse4", infl. = "burlywood3"),
  Disease = c(CD = "blue4", UC = "cadetblue2")
                        )
pheatmap(dists, col = (hmcol),
          annotation_row = annotation_for_heatmap,
          annotation_colors = ann_colors,
          legend = TRUE,
          treeheight_row = 0,
          legend_breaks = c(min(dists, na.rm = TRUE),
                            max(dists, na.rm = TRUE)),
          legend_labels = (c("small distance", "large distance")),
          main = "Clustering heatmap for the calibrated samples")

**Figure 7.**
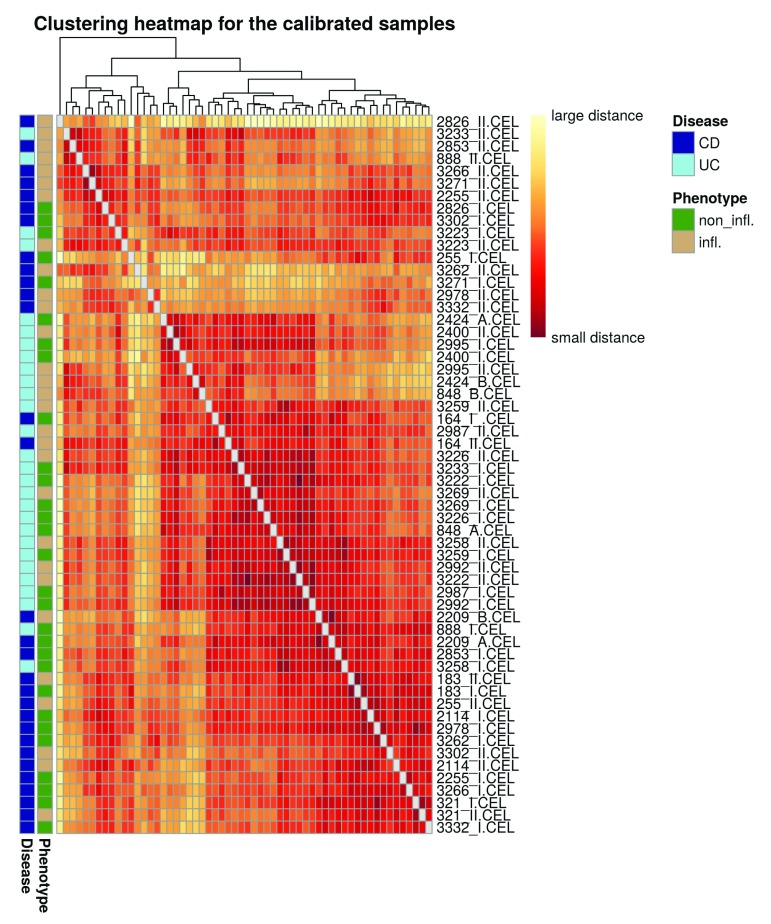
Heatmap of the sample-to-sample distances.

On the heatmap plot ( Figure 7) we also see that the samples do not cluster strongly by tissue, confirming the impression from the PCA plot ( Figure 6) that the separation between the tissues is not perfect. The yellow stripes in the heatmap might correspond to outliers that could potentially be removed: the ones that could be flagged here are 2826_II, 3262_II, 3271_I, 2978_II and 3332_II. 2826_II, 3262_II and 3332_II were found to be outliers in both RLE and heatmap analysis and could therefore potentially be removed; however, in order to stay as close as possible to the original paper, we continue with the complete set of samples. Note again that more elaborate metrics to identify and remove outliers are provided by the *arrayQualityMetrics* package.

## Filtering based on intensity

We now filter out lowly expressed genes. Microarray data commonly show a large numberof probes in the background intensity range. These probes also do not change much across arrays. Hence they combine a low variance with a low intensity. Thus, they could end up being detected as differentially expressed although they are barely above the “detection” limit and are not very informative in general.

We will perform a “soft” intensity based filtering here, since this is recommended by the *limma* ^11, 12^ user guide (a package we will use below for the differential expression analysis).

However, note that a variance based filter might exclude a similar set of probes in practice. For intensity-based filtering, we calculate the row-wise medians from the expression data, as they represent the transcript medians, and assign them to palmieri_medians. From this we create a histogram:

palmieri_medians <- rowMedians(Biobase::exprs(palmieri_eset_norm))

hist_res <- hist(palmieri_medians, 100, col = "cornsilk1", freq = FALSE,
             main = "Histogram of the median intensities",
             border = "antiquewhite4",
             xlab = "Median intensities")

**Figure 8.**
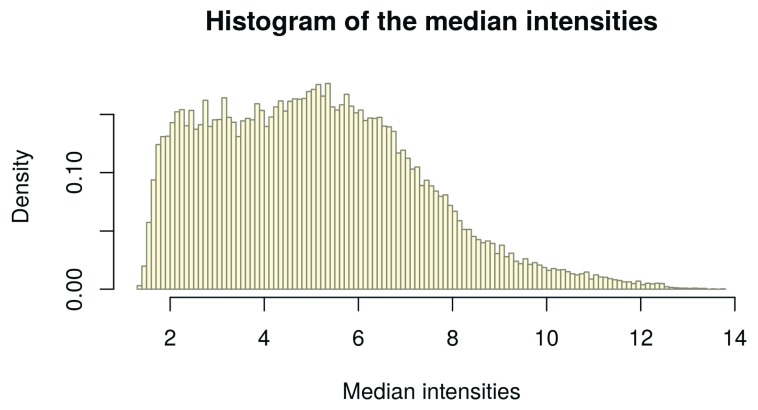
Histogram of the median intensities per gene.

**Figure 9.**
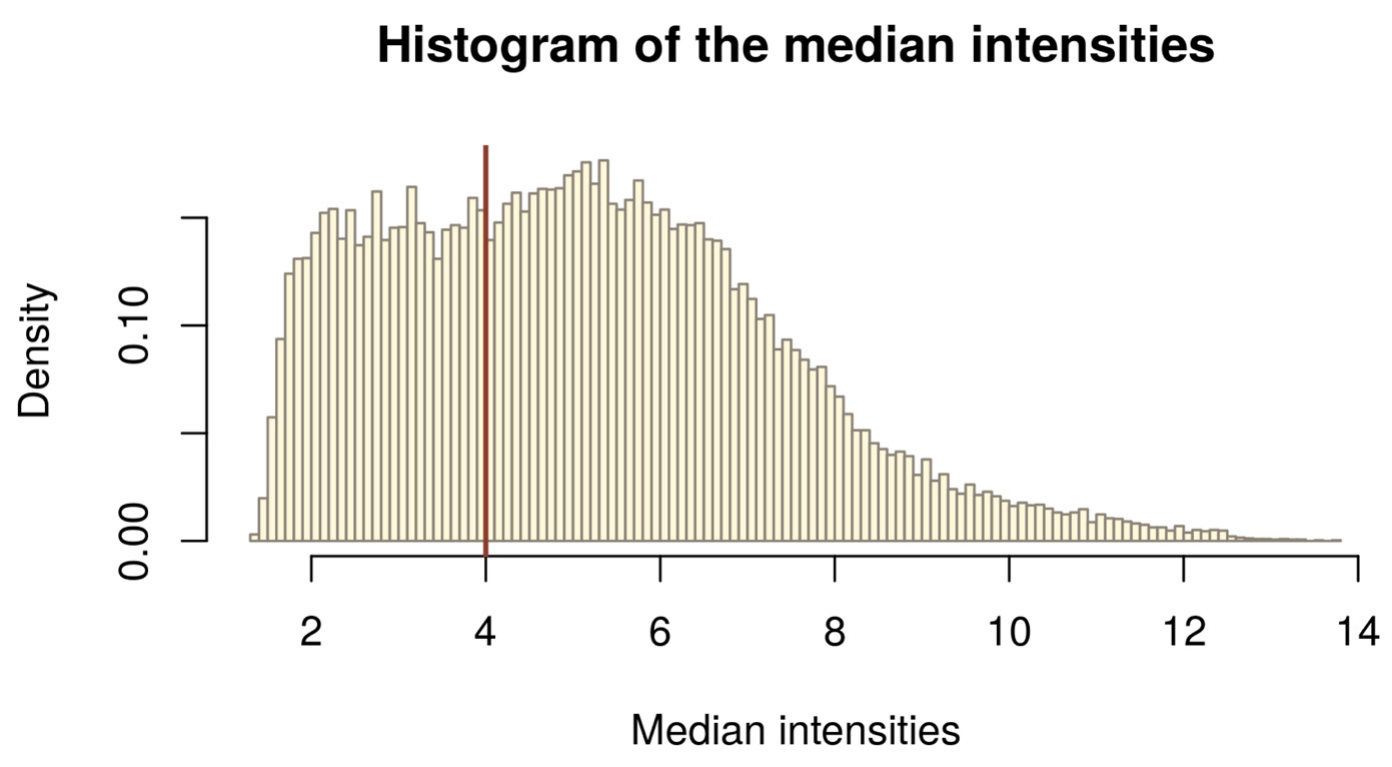
Histogram of the median intensities per gene with manual intensity filtering threshold (red line).

In the histogram of the gene-wise medians ( Figure 8), we can clearly see an enrichment of low medians on the left hand side. These represent the genes we want to filter. In order to infer a cutoff from the data, we inspect the histogram: We visually set a cutoff line man_threshold to the left of the histogram peak in order not to exclude too many genes. In our example, we choose a threshold of 4. We plot the same histogram as before and add the threshold line with the abline() function ( Figure 9):

man_threshold <- 4

hist_res <- hist(palmieri_medians, 100, col = "cornsilk", freq = FALSE,
             main = "Histogram of the median intensities",
             border = "antiquewhite4",
             xlab = "Median intensities")

abline(v = man_threshold, col = "coral4", lwd = 2)

Transcripts that do not have intensities larger than the threshold in at least as many arrays as the smallest experimental group are excluded.

In order to do so, we first have to get a list with the number of samples (=arrays) ( no_of_samples) in the experimental groups:

no_of_samples <-
  table(paste0(pData(palmieri_eset_norm)$Factor.Value.disease., "_",
                    pData(palmieri_eset_norm)$Factor.Value.phenotype.))
no_of_samples

          Crohn’s disease_inflamed colonic mucosa
                                               15
      Crohn’s disease_non-inflamed colonic mucosa
                                               15
       ulcerative colitis_inflamed colonic mucosa
                                               14
   ulcerative colitis_non-inflamed colonic mucosa
                                               14

We now filter out all transcripts that do not have intensities greater than the threshold in at least as many arrays as the smallest experimental group (14) which we define as samples_cutoff.

A function idx_man_threshold is then applied to each row, i.e. to each transcript across all arrays. It evaluates whether the number of arrays where the median intensity passes the threshold ( sum(x > man_threshold)) is greater than the samples_cutoff and returns TRUE or FALSE for each row, i.e. each transcript.

We then create a table of idx_man_threshold to summarize the results and get an overview over how many genes are filtered out. In the last step, we subset our expression set to palmieri_manfiltered and keep the TRUE elements of idx_man_threshold.

samples_cutoff <- min(no_of_samples)

idx_man_threshold <- apply(Biobase::exprs(palmieri_eset_norm), 1,
                              function(x){
                             sum(x > man_threshold) >= samples_cutoff})
                             table(idx_man_threshold)

   idx_man_threshold
   FALSE  TRUE
   10493 22804

palmieri_manfiltered <- subset(palmieri_eset_norm, idx_man_threshold)

## Annotation of the transcript clusters

Before we continue with the linear models for microarrays and differential expression, we first add “feature data”, i.e. annotation information to the transcript cluster identifiers stored in the featureData of our ExpressionSet:

anno_palmieri <- AnnotationDbi::select(hugene10sttranscriptcluster.db,
                                      keys = (featureNames(palmieri_manfiltered)),
                                      columns = c("SYMBOL", "GENENAME"),
                                      keytype = "PROBEID")

anno_palmieri <- subset(anno_palmieri, !is.na(SYMBOL))

We used the function select from *AnnotationDbi* to query the gene symbols and associated short descriptions for the transcript clusters. For each cluster, we added the gene symbol ( SYMBOL) and a short description of the gene the cluster represents ( GENENAME).

In a second step, we filtered out the probes that do not map to a gene, i.e. that do not have a gene symbol assigned.

### Removing multiple mappings

Many transcript-cluster identifiers will map to multiple gene symbols, i.e. they can’t be unambigously assigned.

We compute a summary table in the code below to see how many there are:

anno_grouped <- group_by(anno_palmieri, PROBEID)
anno_summarized <-
  dplyr::summarize(anno_grouped, no_of_matches = n_distinct(SYMBOL))

head(anno_summarized)

   # A tibble: 6 x 2
     PROBEID no_of_matches
     <chr>           <int>
   1 7896742             3
   2 7896754             1
   3 7896759             1
   4 7896761             1
   5 7896779             1
   6 7896798             1

anno_filtered <- filter(anno_summarized, no_of_matches > 1)

head(anno_filtered)

   # A tibble: 6 x 2
     PROBEID no_of_matches
     <chr>           <int>
   1 7896742             3
   2 7896937             3
   3 7896961             3
   4 7897006             2
   5 7897632             3
   6 7897774             2

probe_stats <- anno_filtered

nrow(probe_stats)

   [1] 1771

First, we grouped anno_palmieri by their PROBEID; that way, the subsequent operations are not carried through for each single row, but for each group, i.e. each PROBEID. We then summarized the groups and indicate the number of different genes assigned to a transcript cluster in the column no_of_matches. Finally, we filtered for PROBEIDs with multiple matches, i.e. no_of_matches > 1.

With dim(probe_stats), we could see how many probes have been mapped to multiple genes.

We have close to 2000 transcript clusters that map to multiple gene symbols. It is difficult to decide which mapping is “correct”. Therefore, we exclude these transcript clusters.

We want to remove those probe IDs that match the ones in probe_stats, as those are the probes with multiple mappings. We assign these IDs to the variable ids_to_exclude. Then, we generate palmieri_final, an expression set without the ids_to_exclude.

ids_to_exlude <- (featureNames(palmieri_manfiltered) %in% probe_stats$PROBEID)


table(ids_to_exlude)

   ids_to_exlude
   FALSE  TRUE
   21033  1771

palmieri_final <- subset(palmieri_manfiltered, !ids_to_exlude)

validObject(palmieri_final)

   [1] TRUE

As we have just excluded probe IDs from the assay data, we now have to also exclude them from the feature data anno_palmieri:

head(anno_palmieri)

        PROBEID       SYMBOL
   2657 7896742  LINC00266-1
   2658 7896742       PCMTD2
   2659 7896742    LINC01881
   2664 7896754 LOC100287497
   2665 7896759    LINC01128
   2666 7896761       SAMD11
                                                                            GENENAME
   2657                                 long intergenic non-protein coding RNA 266-1
   2658 protein-L-isoaspartate (D-aspartate) O-methyltransferase domain containing 2
   2659                                  long intergenic non-protein coding RNA 1881
   2664                                                          septin 7 pseudogene
   2665                                  long intergenic non-protein coding RNA 1128
   2666                                     sterile alpha motif domain containing 11

Recall that fData enables us to access the feature data of an expression set. Until now, no feature data whatsoever is stored in the fData(palmieri_final). Only the row names are the row names of the assay data by default, which are the PROBEIDs of the transcripts.

Therefore, we generate a column PROBEID in fData(palmieri_final) and assign the row names of fData(palmieri_final) to it:

fData(palmieri_final)$PROBEID <- rownames(fData(palmieri_final))

Then, we left-join fData(palmieri_final) with anno_palmieri, which already contains the columns “SYMBOL” and “GENENAME”. A left-join keeps the rows and columns of the first argument and adds the corresponding column entries of the second argument:

fData(palmieri_final) <- left_join(fData(palmieri_final), anno_palmieri)

   Joining, by = "PROBEID"

# restore rownames after left_join
rownames(fData(palmieri_final)) <- fData(palmieri_final)$PROBEID

validObject(palmieri_final)

   [1] TRUE

By left-joining with anno_palmieri, we thus add the “SYMBOL” and “GENENAME” columns from anno_palmieri for only the PROBEIDs that are in fData(palmieri_final) and thus get the feature Data for the filtered probes.

By left-joining with anno_palmieri, we thus add the “SYMBOL” and “GENENAME” columns from anno_palmieri for only the PROBEIDs that are in fData(palmieri_final) and thus get the feature Data for the filtered probes.

### Building custom annotations

Alternatively, one can re-map the probes of the array to a current annotation. A workflow to do this for Illumina arrays is given in Arloth *et al*. ^13^. Essentially, the individual probe sequences are re-aligned to an in-silico “exome” that consists of all annotated transcript exons.

In any case, the package *pdInfoBuilder* can be used to build customannotation packages for use with *oligo*. In order to do this, PGF / CLF files (called “Library files” on the Affymetrix website) as well as the probeset annotations are required. The probesets typically represent small stretches of the genome (such as a single exon) and multiple probesets are then used to form a transcript cluster.

The CLF file contains information about the location of individual probes on the array. The PGF file then contains the individual probe sequences and shows the probeset they belong to. Finally, the probeset annotation .csv then contains information about which probesets are used in which transcript cluster. Commonly, multiple probesets are used in one transcript cluster and some probesets are contained in multiple transcript clusters.

## Linear models

In order to analyse which genes are differentially expressed between inflamed and non-inflamed tissue, we have to fit a linear model to our expression data. Linear models are the “workhorse” for the analysis of experimental data. They can be used to analyse almost arbitrarily complex designs, however they also take a while to learn and understand and a thorough description is beyond the scope of this workflow.

Mike Love’s and Michael Irizzary’s genomics class ^14^ is a very good resource, especially the section on interactions and contrasts. It might also be helpful to learn some linear algebra to better understand the concepts here. The Khan Academy offers helpful (and free) online courses.

### Linear models for microarrays

We now apply linear models to microarrays. Specifically, we discuss how to use the *limma* package for differential expression analysis. The package is designed to analyze complex experiments involving comparisons between many experimental groups simultaneously while remaining reasonably easy to use for simple experiments. The main idea is to fit a linear model to the expression data for each gene. Empirical Bayes and other methods are used to borrow information across genes for the residual variance estimation leading to “moderated” *t*-statistics, and stabilizing the analysis for experiments with just a small number of arrays ^12^. Conceptually, the final per gene variance is a mix of a prior variance and the per gene variance.

Typical experimental designs are disussed in chapter 9 of *limma* “User Guide”, which can be found on the Bioconductor landing page of *limma*.

In the following, we use appropriate design and contrast matrices for our linear models and fit a linear model to each gene separately.

### A linear model for the data

For the subsequent linear modelling of the data, we introduce the abbreviations “UC” and “CD” for the disease types, and “non_infl.” and “infl.” for the phenotypes, respectively:

individual <-
  as.character(Biobase::pData(palmieri_final)$Characteristics.individual.)
     
tissue <- str_replace_all(Biobase::pData(palmieri_final)$Factor.Value.phenotype.,
                    " ", "_")
                          
tissue <- ifelse(tissue == "non-inflamed_colonic_mucosa",
                   "nI", "I")
                         
disease <- 
  str_replace_all(Biobase::pData(palmieri_final)$Factor.Value.disease.,
                    " ", "_")
                        
disease <- 
  ifelse(str_detect(Biobase::pData(palmieri_final)$Factor.Value.disease.,
                      "Crohn"), "CD", "UC")

The original paper is interested in changes in transcription that occur between inflamed and adjacent non-inflamed mucosal areas of the colon. This is studied in both inflammatory bowel disease types.

For building our linear model, we have to think about which experimental variables we want to consider. As we want to find differential expression between non-inflamed and inflamed tissue, in principle, those are the only two variables we would have to consider.

However, since we have two arrays per individual patient, we have a “Paired Samples” design (see section 9.4 of the *limma* user guide). This means that the samples might be biased by the person they come from. Whenever a feature in an experimental setup is expected to have a systematic influence on the result, blocking factors on these features should be introduced.

Thus, the first factor we need is a blocking factor for the individuals that will absorb differences in expression between them. Therefore, we block on patients, which means that the patient IDs become variables of the linear model.

Then we create factors that give us the grouping for the tissue types (non-inflamed and inflamed).

Finally, we create two design matrices, one for each of the two diseases as we will analyze them separately in order to follow the analysis strategy of the original paper closely (one could also fit a joint model to the complete data set; however, the two diseases might show very different characteristics so that a joint fit might not be appropriate).

i_CD <- individual[disease == "CD"]
design_palmieri_CD <- model.matrix(~ 0 + tissue[disease == "CD"] + i_CD)
colnames(design_palmieri_CD)[1:2] <- c("I", "nI")
rownames(design_palmieri_CD) <- i_CD


i_UC <- individual[disease == "UC"]
design_palmieri_CD <- model.matrix(~ 0 + tissue[disease == "UC"] + i_UC )
colnames(design_palmieri_UC)[1:2] <- c("I", "nI")
rownames(design_palmieri_UC) <- i_UC

We can inspect the design matrices:

head(design_palmieri_CD[, 1:6])

    
        I nI i_CD183 i_CD2114 i_CD2209 i_CD2255
   164  0  1       0        0        0        0
   164  1  0       0        0        0        0
   183  0  1       1        0        0        0
   183  1  0       1        0        0        0
   2114 0  1       0        1        0        0
   2114 1  0       0        1        0        0


head(design_palmieri_UC[, 1:6])


        I nI i_UC2424 i_UC2987 i_UC2992 i_UC2995
   2400 0  1        0        0        0        0
   2400 1  0        0        0        0        0
   2424 0  1        1        0        0        0
   2424 1  0        1        0        0        0
   2987 0  1        0        1        0        0
   2987 1  0        0        1        0        0

In the design matrix, the rows represent the patient array, and the columns are the variables we include in our linear model. The variables correspond to our blocking factors: there are two for non-inflamed and inflamed tissue, respectively, and one for each patient. “ i_UC2424” for example is the blocking variable of patient 2424; UC stands for the disease the patient is suffering from. For example, the first two rows of the design matrix design_palmieri_CD correspond to the two arrays for individual “164”.

The design matrix entries are 0 or 1 and thus tell us which variables are “active” for which sample:

It can be seen as a switch that turns the single variables “on” (with a 1 at the corresponding position) or “off” (with a 0 at the corresponding position) for each row, i. e. each patient array. If we take a closer look at the single rows, we see that for each sample, there are two “ones” assigned: one to one of the variables “nI” or “I” corresponding to the tissue the array came from, and one to the corresponding patient-specific blocking variable.

Note that in the linear model, individual 164 serves as the baseline for all other individuals and thus isn’t included in the sample variables.

Note that instead of blocking on individuals, it would also be possible to use a “mixed model” approach with the duplicateCorrelation() function from the *limma* package. It has advantages over the “fixed patient effect model” presented here in terms of applicability to more complicated experimental designs, where we want to perform comparisons both within and between the patients (e.g. comparing between the two diseases; “split-plot-designs”).

More information on it can be found in the limma User’s Guide (section 17.3.6). However, the above explained is more intuitive and is therefore used here.

Before heading on to find all differentially expressed genes for both diseases, we will first have a look at how this works in principle for one gene. We will fit the linear model for one gene and run a t-test in order to see whether the gene is differentially expressed or not.

### Analysis of differential expression based on a single gene

For linear model fitting and subsequent testing for differential expression by t-test, we will pick the gene with the PROBEID 8164535. It has the gene symbol *CRAT* and will be named as such in the following code.

***Illustration of the fitted linear model on the *CRAT* gene.*** Before fitting the linear model, we have a look at the expression intensities of this gene for each patient in non-inflamed and inflamed tissue, respectively:

tissue_CD <- tissue[disease == "CD"]
crat_expr <- Biobase::exprs(palmieri_final)["8164535", disease == "CD"]
crat_data <- as.data.frame(crat_expr)
colnames(crat_data)[1] <- "org_value"
crat_data <- mutate(crat_data, individual = i_CD, tissue_CD)

crat_data$tissue_CD <- factor(crat_data$tissue_CD, levels = c("nI", "I"))

ggplot(data = crat_data, aes(x = tissue_CD, y = org_value,
                                group = individual, color = individual)) +
       geom_line() +
       ggtitle("Expression changes for the CRAT gene")

**Figure 10.**
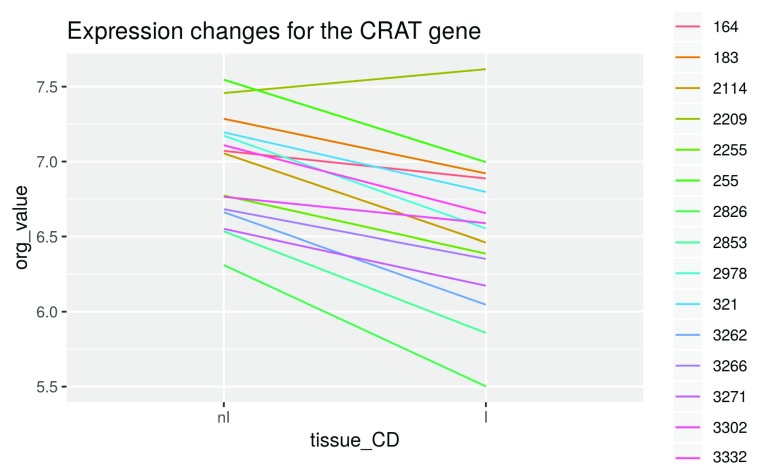
Visualization of expression changes.

We see that overall, this gene is expressed less in inflamed tissue ( Figure 10). We also see that the absolute expression intensities vary greatly between patients. However, we have already taken care of this problem by introducing blocking factors based on the individuals, which allows us to compare the tissues within each individual as represented by the single lines.

If we had not blocked for individuals, the linear model would treat them interchangably and a graphical depiction would only include a single line. Since the individuals haver very different baseline expression levels, this would lead to a very high variance of the estimated fold changes.

We now compute the variable coefficients by fitting a linear model. We get a vector crat_coef with one entry for each variable.

crat_coef <- lmFit(palmieri_final[,disease == "CD"],
                  design = design_palmieri_CD)$coefficients["8164535",]

crat_coef

           I       nI i_CD183 i_CD2114 i_CD2209 i_CD2255  i_CD255 i_CD2826
    6.76669  7.19173 0.12382 -0.22145  0.55759 -0.39905  0.29204 -1.07285
   i_CD2853 i_CD2978 i_CD321 i_CD3262 i_CD3266 i_CD3271 i_CD3302 i_CD3332
   -0.78285 -0.11633 0.01692 -0.62480 -0.46209 -0.61732 -0.30257 -0.09709

In order to now obtain the expression values fitted by the model, we multiply the design matrix with this vector of coefficients crat_coef:

crat_fitted <- design_palmieri_CD %*% crat_coef
rownames(crat_fitted) <- names(crat_expr)
colnames(crat_fitted) <- "fitted_value"

crat_fitted
	

                 fitted_value
   164_I_.CEL	      7.192
   164_II.CEL	      6.767
   183_I.CEL	      7.316
   183_II.CEL	      6.891
   2114_I.CEL	      6.970
   2114_II.CEL	      6.545
   2209_A.CEL	      7.749
   2209_B.CEL	      7.324
   2255_I.CEL	      6.793
   2255_II.CEL	      6.368
   255_I.CEL	      7.484
   255_II.CEL	      7.059
   2826_I.CEL	      6.119
   2826_II.CEL	      5.694
   2853_I.CEL	      6.409
   2853_II.CEL	      5.984
   2978_I.CEL	      7.075
   2978_II.CEL	      6.650
   321_I.CEL 	      7.209
   321_II.CEL	      6.784
   3262_I.CEL	      6.567
   3262_II.CEL	      6.142
   3266_I.CEL	      6.730
   3266_II.CEL	      6.305
   3271_I.CEL	      6.574
   3271_II.CEL	      6.149
   3302_I.CEL	      6.889
   3302_II.CEL	      6.464
   3332_I.CEL	      7.095
   3332_II.CEL	      6.670

Recall that for every row in the design matrix (i.e. every patient sample) only the variables with a 1 in the design matrix are taken into account for calculating the fitted expression value.

This means that as output of the multiplication, we get a vector crat_fitted whose entries are the sum of relevant variable coefficients for each sample, respectively.

For example, the fitted value for patient sample 2114_I.CEL is 6.9703: it is the sum of the respective activated variable coefficients “nI” (7.1917) and “i_CD2114” (-0.2215).

Let’s visualize the difference between non-inflamed and inflamed tissue again after fitting:

crat_data$fitted_value <- crat_fitted
ggplot(data = crat_data, aes(x = tissue_CD, y = fitted_value,
                                group = individual, color = individual)) +
       geom_line() +
       ggtitle("Fitted expression changes for the CRAT gene")

**Figure 11.**
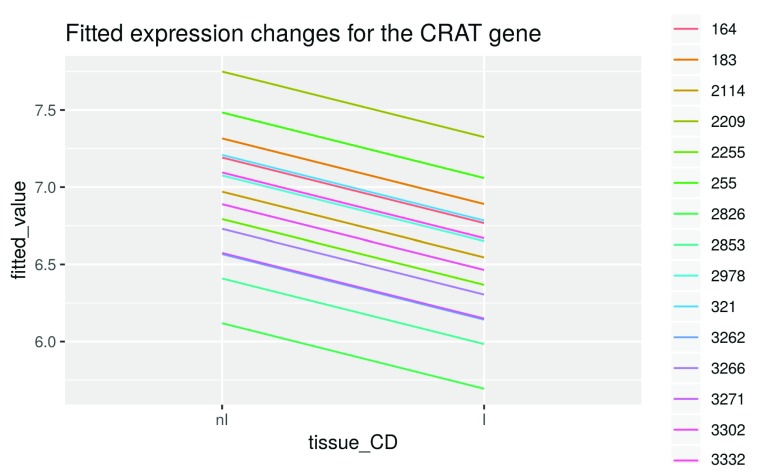
Expression changes for the CRAT gene.

Note that the difference of the fitted expression values between inflamed and non-inflamed samples of one patient is the same for all patients and is determined by the difference between the variable coefficients of I (6.7667) and nI (7.1917), which is -0.425 ( Figure 11).

This is the case because the same blocking variable is activated by the design matrix for both samples from a single patient, leading to a comparison within patients only. These blocking variables correct the fitted tissue specific expression values towards the expression levels of the individual patients. Therefore the final estimate is like an average of all the within-individual differences.

The “difference” between non-inflamed and inflamed tissue of -0.425 is actually a log2 fold change, as our expression data is on the log2 scale. -0.425 therefore is our log2 fold change for the CRAT gene.

***Differential expression analysis of the CRAT gene.*** In order to test whether the gene is differentially expressed or not, a *t*-test with the null hypothesis that there is no difference in the expression between non-inflamed and inflamed tissue is carried out. Our blocking design is conceptually similar to a paired t-test for which the statistic is given by:

$$t=\frac{\bar{d}}{s/\sqrt{n}}$$

Where, *d̅* is the mean difference in expression values between the individuals. The paired t-test computes the variance *s* ^2^ from the paired differences. This is lower than the variance of a standard t-test and thus the paired t-test has higher power as long as the expression values for the same individual are correlated ( see e.g. the article on Wikipedia).

We thus have improved the power of the ordinary *t*-test by reducing the variance via blocking on individuals.

We now conduct the *t*-test on the linear model in order to find out whether the difference between non-inflamed and inflamed tissue differs significantly from 0:

crat_noninflamed <- na.exclude(crat_data$org_value[tissue == "nI"])
crat_inflamed <- na.exclude(crat_data$org_value[tissue == "I"])
res_t <- t.test(crat_noninflamed ,crat_inflamed , paired = TRUE)
res_t

    Paired t-test
    data:  crat_noninflamed and crat_inflamed
    t = 6.8, df = 14, p-value = 8e-06
    alternative hypothesis: true difference in means is not equal to 0
    95 percent confidence interval:
     0.2919 0.5581
    sample estimates:
    mean of the differences
                      0.425

We get a low p-value close to 0 and thus can conclude that the *CRAT* gene is differentially expressed between non-inflamed and inflamed tissue.

Note that the p-value isn’t exactly the same one as below when analyzing the differential expression of all genes. This is due to the variance moderation performed by *limma*.

### Contrasts and hypotheses tests

We now fit the linear model for all genes and define appropriate contrasts to test hypotheses of interest.

We want to compare the inflamed to the non-inflamed tissue. Thus, we create a contrast matrix consisting of only one contrast “I-nI”: *limma*’s function makeContrasts creates this matrix from a symbolic description of the contrast of interest.

We now fit a linear model to our data and apply the contrasts.fit() function to it in order to find genes with significant differential expression between non-inflamed and inflamed tissue:

contrast_matrix_CD <- makeContrasts(I-nI, levels = design_palmieri_CD)

palmieri_fit_CD <- eBayes(contrasts.fit(lmFit(palmieri_final[,disease == "CD"],
                                    design = design_palmieri_CD),
                                contrast_matrix_CD))

contrast_matrix_UC <- makeContrasts(I-nI, levels = design_palmieri_UC)

palmieri_fit_UC <- eBayes(contrasts.fit(lmFit(palmieri_final[,disease == "UC"],
                                    design = design_palmieri_UC),
                                contrast_matrix_UC))

We applied the empirical Bayes variance moderation method to the model via the eBayes() function, which computes moderated *t*-statistics. In microarray analysis, the number of arrays often is quite small, and thus variance estimation is difficult. Using a combination of the per-gene-variance and a prior variance we can improve the variance estimate, hence the term “moderation”. “Empirical Bayes” means that the prior is estimated from the data.

The result of the eBayes() step is that the individual variances are shrunken towards the prior value.

### Extracting results

**Figure 12.**
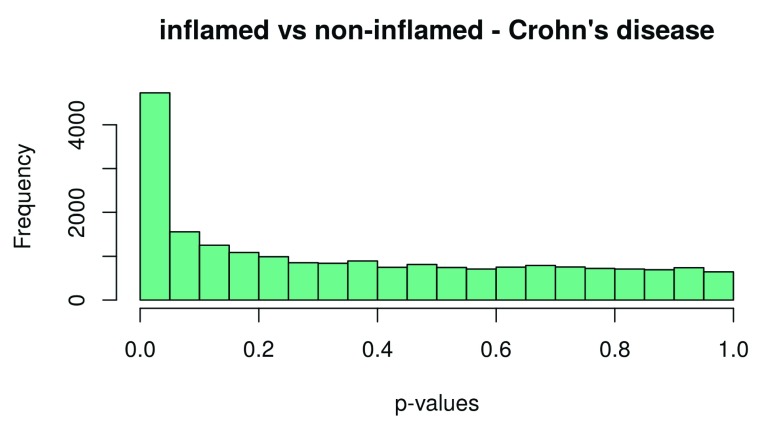
Histogram of the p–values for Crohn’s disease.

**Figure 13.**
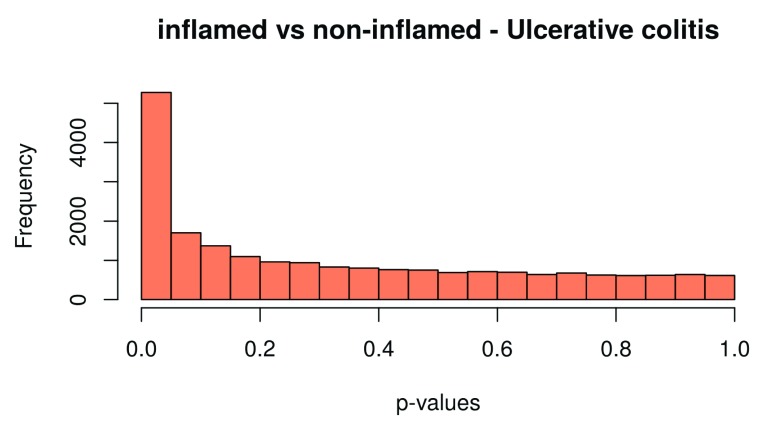
Histogram of the p–values for ulcerative colitis.

Finally, we extract the number of differentially expressed genes. Results can be extracted by use of the topTable function. We extract the results for both Crohn’s disease and ulcerative colitis, and the results are sorted by their absolute *t*-statistics. As a diagnostic check, we also plot the p-value histogram ( Figure 12 and Figure 13): We expect a uniform distribution for the p-values that correspond to true null hypotheses, while a peak near zero shows an enrichment for low p-values corresponding to differentially expressed (DE) genes.

Note that if the p-value distribution for a dataset is very different from the ones in the histograms below, this might lead to quality loss in the subsequent analysis. Reasons for a divergent p-value-distribution might be batch effects or a lack of consideration of other blocking factors in the design matrix. Thus, if the p-value is not as expected, try to include possible blocking factors and batches and rerun the analysis. If this does not help, empirical Bayes / null estimation methods for multiple testing are useful.

A good starting point to learn about these methods is the article on false discovery rate estimation by Korbininan Strimmer ^15^ and chapter 1–6 of Efron’s book on Large-Scale Inference ^16^, as well as the blog-post on “How to interpret a p-value histogram” by David Robinson ^17^.

table_CD <- topTable(palmieri_fit_CD, number = Inf)
head(table_CD)

           PROBEID  SYMBOL                                     GENENAME   logFC
   7928695 7928695 FAM213A family with sequence similarity 213 member A -0.5990
   8123695 8123695    ECI2                  enoyl-CoA delta isomerase 2 -0.4855
   8164535 8164535    CRAT                carnitine O-acetyltransferase -0.4250
   8009746 8009746 SLC16A5            solute carrier family 16 member 5 -0.5182
   7952249 7952249    USP2               ubiquitin specific peptidase 2 -0.8484
   8105348 8105348    GPX8          glutathione peroxidase 8 (putative)  0.8312
           AveExpr      t   P.Value adj.P.Val     B
   7928695   7.739 -7.059 1.383e-06   0.02092 5.305
   8123695   6.876 -6.317 5.907e-06   0.02092 4.028
   8164535   6.732 -6.230 7.037e-06   0.02092 3.872
   8009746   5.562 -6.206 7.386e-06   0.02092 3.829
   7952249   5.606 -6.203 7.429e-06   0.02092 3.824
   8105348   5.301  6.074 9.656e-06   0.02092 3.590

hist(table_CD$P.Value, col = brewer.pal(3, name = "Set2")[1],
      main = "inflamed vs non-inflamed - Crohn’s disease", xlab = "p-values")

table_UC <- topTable(palmieri_fit_UC, number = Inf)
head(table_UC)

           PROBEID  SYMBOL                            GENENAME   logFC AveExpr
   8003875 8003875   SPNS2          sphingolipid transporter 2  0.7412   6.478
   8082012 8082012 SLC15A2   solute carrier family 15 member 2  0.8061   5.319
   7952290 7952290  TRIM29      tripartite motif containing 29  1.0140   5.855
   7961693 7961693    LDHB             lactate dehydrogenase B  0.3968   9.534
   8072015 8072015    GRK3 G protein-coupled receptor kinase 3  0.4713   5.584
   8096070 8096070    BMP3        bone morphogenetic protein 3 -1.6961   6.420
                t   P.Value adj.P.Val     B
   8003875  7.801 4.553e-07  0.003983 6.441
   8082012  7.744 5.033e-07  0.003983 6.352
   7952290  7.482 8.009e-07  0.003983 5.937
   7961693  7.401 9.265e-07  0.003983 5.806
   8072015  7.308 1.097e-06  0.003983 5.654
   8096070 -7.289 1.136e-06  0.003983 5.623

hist(table_UC$P.Value, col = brewer.pal(3, name = "Set2")[2],
      main = "inflamed vs non-inflamed - Ulcerative colitis", xlab = "p-values")

### Multiple testing FDR, and comparison with results from the original paper

In the original paper, a p-value of 0.001 was used as a significance cutoff. Using this we get 947 genes identified as differentially expressed for UC:

nrow(subset(table_UC, P.Value < 0.001))

   [1] 947

However, it is impossible to determine a precise bound on the number of false positive genes in this list. All that we can say using p-values is that we have at most 21033 (total number of tests) * 0.001 = 21.033 false positive genes in our list. Therefore, by choosing a p-value cutoff of 0.001, as much as 2.22% of our genes identified as differentially expressed might be false positives.

Thus, we can see that the “raw” p-values are very “liberal” when looking at many tests simultaneously. We therefore need error rates adapted to the multiple testing situation. By far the most popular one in molecular biology is the **false discovery rate** or FDR for short. It is the percentage of false positives among all positives. As we have seen, the FDR of our genes list using a simple p-value cutoff might be quite high.

On the other hand, we can see a clear peak in the p-value histogram ( Figure 13), caused by the differentially expressed genes. There we expect the actual FDR of our list to be lower.

The FDR at a given cutoff is given by the “adjusted” p-value in the results table.



tail ( subset (table_UC, P.Value < 0.001 ))
                    




                                   PROBEID   SYMBOL
   7915640 7915640   EIF2B3
   7894577 7894577     <NA>
   7897877 7897877 TNFRSF1B
   8142671 8142671     WASL
   7941946 7941946   NDUFV1
   8140371 8140371 TMEM120A
                                                            GENENAME   logFC
   7915640 eukaryotic translation initiation factor 2B subunit gamma  0.2795
   7894577                                                      <NA>  0.4048
   7897877                           TNF receptor superfamily member  1B 0.3564
   8142671                             Wiskott-Aldrich syndrome like -0.4281
   7941946            NADH:ubiquinone oxidoreductase core subunit V1 -0.3832
   8140371                                transmembrane protein 120A -0.3657
           AveExpr      t   P.Value adj.P.Val       B
   7915640   6.271  3.957 0.0009867   0.02203 -0.6478
   7894577   5.598  3.956 0.0009892   0.02205 -0.6501
   7897877   6.656  3.956 0.0009899   0.02205 -0.6508
   8142671   7.907 -3.955 0.0009906   0.02205 -0.6515
   7941946   8.867 -3.952 0.0009976   0.02218 -0.6581
   8140371   6.519 -3.951 0.0009990   0.02219 -0.6594
                    


The adjusted p-value for a raw p-value of 0.001 in the table is 0.0222, which is an order of magnitude lower than the FDR we can infer from p-values alone.

So although this is not recommended in general, we also use a p-value cutoff at 0.001 in the following in order to be able to compare our workflow results to the paper results.

The paper results can be downloaded as excel files from http://links.lww.com/IBD/A795 and should be saved as an .xlsx file named palmieri_DE_res.xlsx in your working directory.

Note that while the paper uses p-value cutoffs, it also reports the corresponding FDRs (just as we did for the UC data here).

For a p-value cutoff of 0.001, the corresponding FDRs are 0.05 in Crohn’s disease and 0.02 in ulcerative colitis. There are four tables in total, giving the list of up and downregulated genes in CD and UC, respectively. We calculate the overlap between our results and the ones from the paper as the ratio of the genes that were found in both analyses and the genes that were only found in the paper.

We also calculate the total number of diffentially expressed genes that we find in our workflow analysis.

fpath <- system.file("extdata", "palmieri_DE_res.xlsx", package = "maEndToEnd")
palmieri_DE_res <- sapply(1:4, function(i) read.xlsx(cols = 1, fpath,
                                                               sheet = i, startRow = 4))

names(palmieri_DE_res) <- c("CD_UP", "CD_DOWN", "UC_UP", "UC_DOWN")
palmieri_DE_res <- lapply(palmieri_DE_res, as.character)
paper_DE_genes_CD <- Reduce("c", palmieri_DE_res[1:2])
paper_DE_genes_UC <- Reduce("c", palmieri_DE_res[3:4])

overlap_CD <- length(intersect(subset(table_CD, P.Value < 0.001)$SYMBOL,
                                   paper_DE_genes_CD)) / length(paper_DE_genes_CD)


overlap_UC <- length(intersect(subset(table_UC, P.Value < 0.001)$SYMBOL,
                                   paper_DE_genes_UC)) / length(paper_DE_genes_UC)
overlap_CD

   [1] 0.6443

overlap_UC

   [1] 0.6731

total_genenumber_CD <- length(subset(table_CD, P.Value < 0.001)$SYMBOL)
total_genenumber_UC <- length(subset(table_UC, P.Value < 0.001)$SYMBOL)

total_genenumber_CD

   [1] 575

total_genenumber_UC

   [1] 947

We find 575 (CD) and 947 (UC) differentially expressed genes (“DE-genes”).

In the paper, 298 (CD) and 520 (UC) DE-genes were found for the two diseases at the same cutoff. This higher number of DE-genes identified is probably due to the increased power of the blocking according to the individuals and the moderated variance estimation that *limma* performs.

We see that we get a moderate overlap of 0.6443 for CD and 0.6731 for UC, showing that both analyses lead to somewhat comparable results.

### Visualization of DE analysis results - volcano plot

For a visualization of the differentially expressed genes, we create a volcano plot, which is commonly used to summarize the results of a differential expression analysis in a single figure.

For a better overview, we only show gene symbols of genes with a fold change greater than 1, which we define in the volcano_names object. The highlight option in the volcanoplot function is set to 100 and thus only labels the 100 genes with the lowest p-values.

volcano_names <- ifelse(abs(palmieri_fit_CD$coefficients)>=1,
                           palmieri_fit_CD$genes$SYMBOL, NA)
              
volcanoplot(palmieri_fit_CD, coef = 1L, style = "p-value", highlight = 100,
             names = volcano_names,
             xlab = "Log2 Fold Change", ylab = NULL, pch=16, cex=0.35)
             


**Figure 14.**
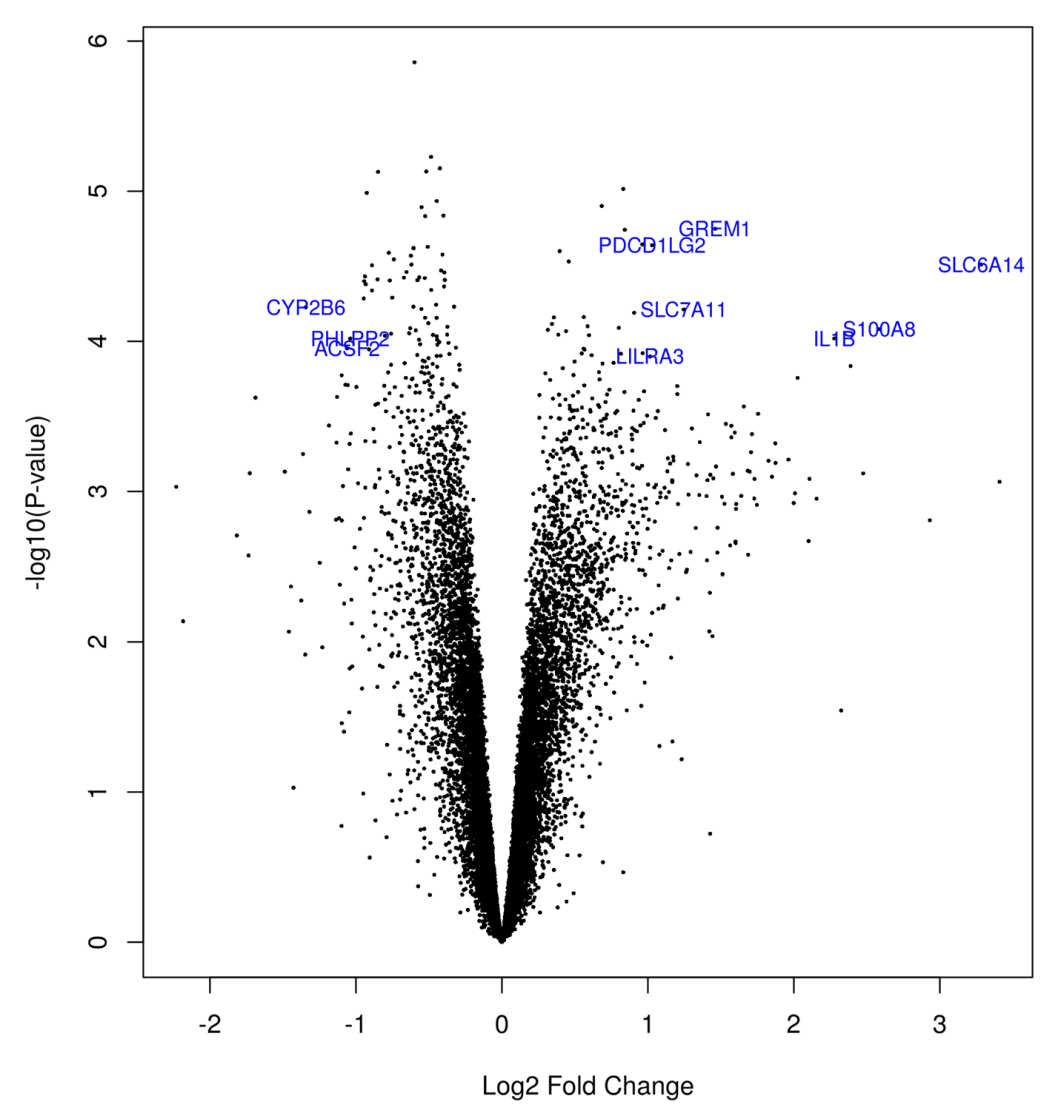
Volcano plot of the DE-genes.

We can now do a little research on the biological function of genes that show a high foldchange, for example the gene with the symbol S100A8 on the right hand side of the plot ( Figure 14). If we search for this gene symbol on genecards.org, we find that it encodes for a protein that builds a pro-inflammatory complex in association with another protein.

## Gene ontology (GO) based enrichment analysis

As discussed above, it is recommended to use an FDR cutoff in differential expression analysis rather than a p-value cutoff, since this way you control an explicitly defined error rate and the results are easier to interpret and to compare. For the following enrichment analysis, we create tables with differentially expressed genes for CD and UC, respectively, and choose an FDR cutoff of 10%. Here, we focus on the CD subset of the data.

DE_genes_CD <- subset(table_CD, adj.P.Val < 0.1)$PROBEID



We can now try to characterize the identified differentially expressed genes more in detail by performing a GO enrichment analysis. Essentially the gene ontology ( http://www.geneontology.org/) is a hierarchically organized collection of functional gene sets ^18– 20^.

### Matching the background set of genes

The function genefinder from the *genefilter* package ^21^ will be used to find a background set of genes that are similar in expression to the differentially expressed genes. We then check whether the background has roughly the same distribution of average expression strength as the foreground.

We do this in order not to select a biased background since the gene set testing is performed by a simple Fisher test on a 2x2 table. Note that this approach is very similar to commonly used web tools like GOrilla ^22^.

For every differentially expressed gene, we try to find genes with similar expression with genefinder. The genefinder function returns a list with two elements for each gene: one with the indices of the background genes found and one with the distances to the DE-genes:

back_genes_idx <- genefilter::genefinder(palmieri_final,
                                             as.character(DE_genes_CD),
                                             method = "manhattan", scale = "none")
                     

We have to extract the PROBEIDs, which correspond to the indices. We do that by using the sapply function, which gives us a single matrix with the DE-genes as column names and the PROBEIDs of the corresponding background genes in the cells below:

back_genes_idx <- sapply(back_genes_idx, function(x)x$indices)



We then create a vector back_genes containing all background gene PROBEIDs:

In order to eliminate foreground genes, i.e. DE-genes, from the back_genes set, we use the setdiff function. It returns all elements from the first argument ( back_genes) that are not part of the second argument ( DE_genes_CD). With the intersect function, we verify that we were successful: it should return 0, as there shouldn’t be any intersect anymore between back_genes and DE_genes_CD:

back_genes <- featureNames(palmieri_final)[back_genes_idx]
back_genes <- setdiff(back_genes, DE_genes_CD)


intersect(back_genes, DE_genes_CD)

   character(0)

length(back_genes)

   [1] 9756

**Figure 15.**
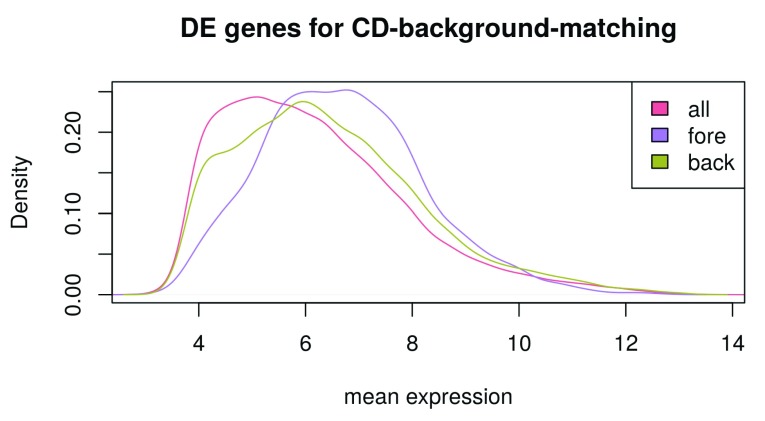
Selecting a background set of genes for the gene ontology analysis.

We create a multidensity plot with mean expression on the x-axis and curves for all genes, foreground genes and background genes, respectively ( Figure 15). We want to see whether the background genes show a plot similar to the foreground genes so that the background is not biased for the gene enrichment analysis:

multidensity(list(
         all = table_CD[,"AveExpr"] ,
         fore = table_CD[DE_genes_CD , "AveExpr"],
         back = table_CD[rownames(table_CD) %in% back_genes, "AveExpr"]),
         col = c("#e46981", "#ae7ee2", "#a7ad4a"),
      xlab = "mean expression",
    main = "DE genes for CD-background-matching")

When comparing the “background gene” curve to the “foreground gene” curve, we see a similar curve shape, indicating a sensible background matching ( Figure 15). Note that the right-shift of the “foreground-gene” curve in comparison to the “background-gene” curve indicates that DE-genes are generally very highly expressed, so that it wasn’t possible to find background-genes with exactly equal overall expression distribution.

The “all gene” curve has the leftmost curve maximum; this can be explained by a high number of lowly expressed genes in all samples and shows that a background matching is sensible in order to avoid biases.

For the actual testing of which GO gene sets are enriched in inflamed tissue, we use the *topGO* package which implements a nice interface to Fisher testing and also has additional algorithms taking the GO structure into account, by e.g. only reporting the most specific gene set in the hierarchy ^23^.

The GO has three top ontologies: Cellular component (CC), biological processes (BP), and molecular function (MF). For illustrative purposes we limit ourselves to the BP category here.

### Running topGO

topGO requires a topGOdata object containing the necessary information for the analysis. We follow the steps described in the topGO vignettes: First, we will create a named vector all_genes with all genes to be analyzed, i.e. DE-genes and background genes:

gene_IDs <- rownames(table_CD)
in_universe <- gene_IDs %in% c(DE_genes_CD, back_genes)
in_selection <- gene_IDs %in% DE_genes_CD

all_genes <- in_selection[in_universe]
all_genes <- factor(as.integer(in_selection[in_universe]))
names(all_genes) <- gene_IDs[in_universe]

The following steps were carried through:

1. we created an in_universe vector by using the %in% matching function. We want to know which elements from gene_IDs are also contained in DE_genes_CD and back_genes, as the latter two are our gene universe we use for enrichment analysis. We got a vector in_universe with the length of gene_IDs that has the entry TRUE when the corresponding gene in gene_IDs could be also found in DE_genes_CD or back_genes, and FALSE otherwise.
2. We did the same for our DE-genes and call this vector in_selection.
3. We created the all_genes vector:
  a) First, we selected all the elements from in_selection that are TRUE in in_universe by applying all_genes <- in_selection[in_universe].
  b) Then, we converted the elements in all_genes from TRUE and FALSE to 0 and 1 by converting the vector to an integer vector. This way, each element in the vector is a 0 if the corresponding gene is a background gene and a 1 if the corresponding gene is a DE-gene. Also, we converted the vector to a factor.
  c) We named the vector elements with the corresponding gene_IDs.

We now initialize the *topGO* data set, using the GO annotations contained in the annotation data base for the chip we are using. The nodeSize parameter specifies a minimum size of a GO category we want to use: i.e. here, categories with less than 10 genes are not included in the testing.

top_GO_data <- new("topGOdata", ontology = "BP", allGenes = all_genes,
 nodeSize = 10, annot = annFUN.db, affyLib = "hugene10sttranscriptcluster.db")

Now the tests can be run. *topGO* offers a wide range of options, for details see the paper ^23^ or the package vignette.

We run two common tests: an ordinary Fisher test for every GO category, and the “elim” algorithm, which tries to incorporate the hierarchical structure of the GO and tries to “decorrelate” it in order to report the most specific significant term in the hierarchy.

The algorithm starts processing the nodes / GO categories from the highest (bottommost) level and then iteratively moves to nodes from a lower level. If a node is scored as significant, all of its genes are marked as removed in all ancestor nodes. This way, the “elim” algorithm aims at finding the most specific node for every gene.

The test uses a 0.01 p-value cutoff by default.

result_top_GO_elim <-
  runTest(top_GO_data, algorithm = "elim", statistic = "Fisher")
result_top_GO_classic <-
  runTest(top_GO_data, algorithm = "classic", statistic = "Fisher")

We can now inspect the results. We look at the top 100 GO categories according to the “Fisher elim” algorithm. The function GenTable produces a table of significant GO categories, the function printGenes gives genes annotated to them; the significant ones are denoted with a “2” in the “raw p-value” column, the non-significant ones with a “1”. We therefore select raw p-value == 2.

Note that we do not get the actual p-values here because our all_genes vector doesn’t contain this information; it only tells us whether a gene is differentially expressed or not.

res_top_GO <- GenTable(top_GO_data, Fisher.elim = result_top_GO_elim,
         Fisher.classic = result_top_GO_classic,
         orderBy = "Fisher.elim" , topNodes = 100)

genes_top_GO <- printGenes(top_GO_data, whichTerms = res_top_GO$GO.ID,
     chip = "hugene10sttranscriptcluster.db", geneCutOff = 1000)

res_top_GO$sig_genes <- sapply(genes_top_GO, function(x){
                  str_c(paste0(x[x$’raw p-value’ == 2, "Symbol.id"],";"),
                         collapse = "")
    })

head(res_top_GO[,1:8], 20)

           GO.ID                                        Term Annotated Significant
   1  GO:0032496              response to lipopolysaccharide       223          96
   2  GO:0006954                       inflammatory response       442         193
   3  GO:0051897 positive regulation of protein kinase B ...       100          49
   4  GO:0050900                         leukocyte migration       282         134
   5  GO:0030335       positive regulation of cell migration       318         130
   6  GO:0006911                    phagocytosis, engulfment        54          29
   7  GO:0030198           extracellular matrix organization       223          96
   8  GO:0070098        chemokine-mediated signaling pathway        45          25
   9  GO:0070374 positive regulation of ERK1 and ERK2 cas...       144          57
   10 GO:0007186 G-protein coupled receptor signaling pat...       441         146
   11 GO:0048661 positive regulation of smooth muscle cel...        57          32
   12 GO:0030574                  collagen catabolic process        48          25
   13 GO:0002675 positive regulation of acute inflammator...        20          14
   14 GO:0030593                       neutrophil chemotaxis        54          32
   15 GO:0042493                            response to drug       566         177
   16 GO:0001937 negative regulation of endothelial cell ...        30          18
   17 GO:0016525         negative regulation of angiogenesis        70          32
   18 GO:0030449         regulation of complement activation        44          23
   19 GO:0006067                   ethanol metabolic process        12          10
   20 GO:0033540 fatty acid beta-oxidation using acyl-CoA...        12          10
      Expected Rank in Fisher.classic Fisher.elim Fisher.classic
   1     49.11                     56     3.1e-10        1.0e-12
   2     97.34                      1     1.0e-09        1.8e-25
   3     22.02                    115     2.2e-09        2.2e-09
   4     62.10                      6     1.3e-07        6.6e-22
   5     70.03                     39     3.2e-07        1.2e-14
   6     11.89                    205     3.5e-07        3.5e-07
   7     49.11                     57     7.7e-07        1.0e-12
   8      9.91                    231     9.7e-07        9.7e-07
   9     31.71                    242     1.3e-06        1.3e-06
   10    97.12                    150     2.6e-06        2.6e-08
   11    12.55                    149     4.3e-06        2.1e-08
   12    10.57                    283     4.7e-06        4.7e-06
   13     4.40                    290     6.0e-06        6.0e-06
   14    11.89                    125     7.1e-06        3.3e-09
   15   124.65                    171     7.5e-06        8.8e-08
   16     6.61                    297     7.6e-06        7.6e-06
   17    15.42                    303     8.6e-06        8.6e-06
   18     9.69                    311     1.0e-05        1.0e-05
   19     2.64                    318     1.1e-05        1.1e-05
   20     2.64                    319     1.1e-05        1.1e-05

### Visualization of the GO-analysis results

A graph of the results can also be produced. Here we visualize the three most significant nodes according to the Fisher elim algorithm in the context of the GO hierarchy.

showSigOfNodes(top_GO_data, score(result_top_GO_elim), firstSigNodes = 3,
                 useInfo = ’def’)

**Figure 16.**
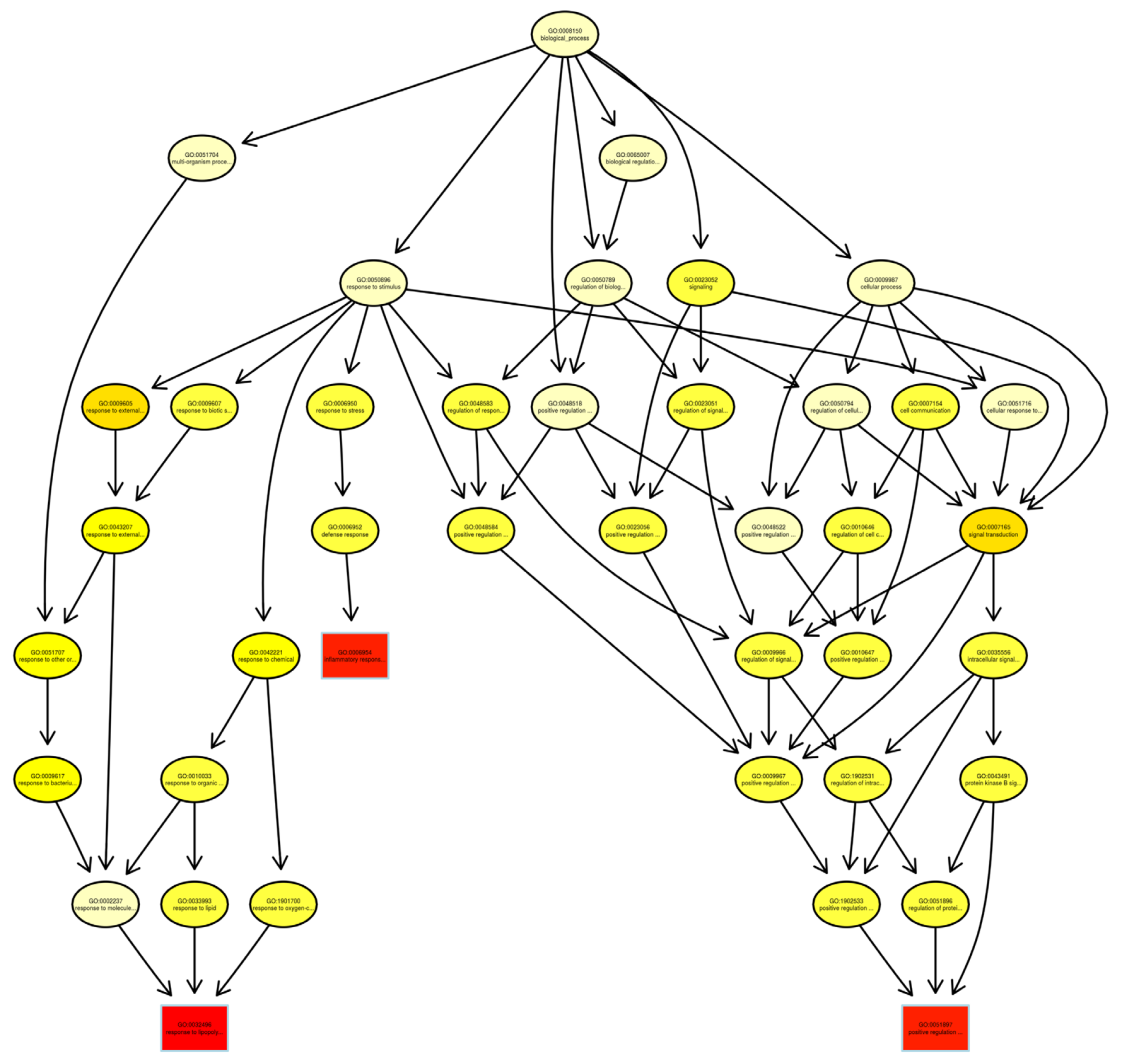
Significantly enriched GO nodes in the GO hierarchy.

We can see that indeed GO categories related to inflammation, signalling and immune response come up as significant ( Figure 16) Gene set enrichment analysis has been a field of very extensive research in bioinformatics. For additional approaches see the *topGO* vignette and the references therein and also in the GeneSetEnrichment view.

## A pathway enrichment analysis using reactome

The package *ReactomePA* offers the possibility to test enrichment of specific pathways using the free, opensource, curated and peer reviewed Reactome pathway database ^24, 25^. The package requires entrez identifiers, so we convert our PROBEIDs (transcript cluster identifiers) to entrez identifiers using the function mapIDs from the package *AnnotationDbi*. This will create a named vector that maps the PROBEIDs to the entrez ones, with the PROBEIDs as names and the entrez ids as vector elements.

entrez_ids <- mapIds(hugene10sttranscriptcluster.db,
       keys = rownames(table_CD),
       keytype = "PROBEID",
       column = "ENTREZID")



We can now run the enrichment analysis that performs a statistical test based on the hypergeoemtric distribution that is the same as a one sided Fisher-test, which *topGO* calls “Fisher-classic”. Details can be found in the vignette of the *DOSE* package ^26^.

reactome_enrich <- enrichPathway(gene = entrez_ids[DE_genes_CD],
                                    universe = entrez_ids[c(DE_genes_CD,
                                                               back_genes)],
                                    organism = "human",
                                    pvalueCutoff = 0.05,
                                    qvalueCutoff = 0.9,
                                    readable = TRUE)

reactome_enrich@result$Description <- paste0(str_sub(
                                        reactome_enrich@result$Description, 1, 20),
                                        "...")
                         
head(summary(reactome_enrich))[1:6]


                            ID             Description GeneRatio  BgRatio
   R-HSA-8978868 R-HSA-8978868 Fatty acid metabolis...   54/1380 104/5934
   R-HSA-6785807 R-HSA-6785807 Interleukin-4 and 13...   41/1380  77/5934
   R-HSA-6783783 R-HSA-6783783 Interleukin-10 signa...   22/1380  31/5934
   R-HSA-556833   R-HSA-556833 Metabolism of lipids...  154/1380 454/5934
   R-HSA-380108   R-HSA-380108 Chemokine receptors ...   19/1380  26/5934
   R-HSA-1474244 R-HSA-1474244 Extracellular matrix...   74/1380 188/5934
                    pvalue  p.adjust
   R-HSA-8978868 1.463e-10 1.494e-07
   R-HSA-6785807 9.450e-09 4.824e-06
   R-HSA-6783783 2.208e-08 7.516e-06
   R-HSA-556833  4.904e-08 1.252e-05
   R-HSA-380108  9.824e-08 2.006e-05
   R-HSA-1474244 3.898e-07 6.633e-05

Note that we trimmed pathway names to 20 characters.

### Visualizing the reactome based analysis results

**Figure 17.**
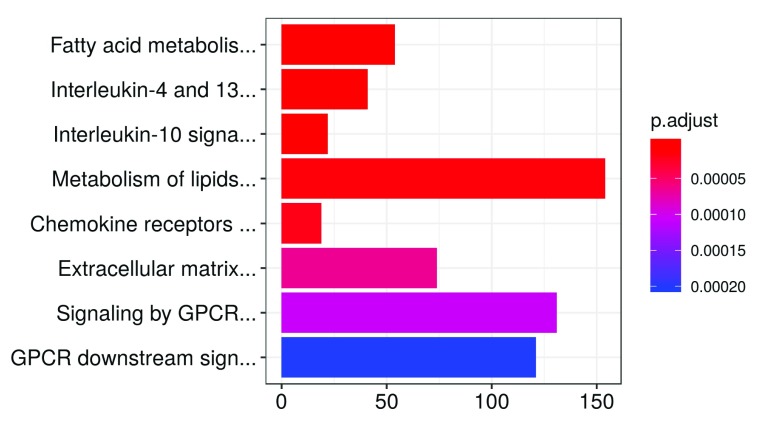
Enriched Reactome pathways and their p–values as a bar chart.

The top pathways can be displayed as a bar chart that displays all categories with a p-value below the specified cutoff (Figure 17).

barplot(reactome_enrich)

The “enrichment map” from the package *enrichplot* displays the results of the enrichment analysis as a graph, where the color represents the p-value of the pathway and the edge-thickness (that is the line connecting two pathways) is proportional to the number of overlapping genes between two pathways.

emapplot(reactome_enrich, showCategory = 10)


**Figure 18.**
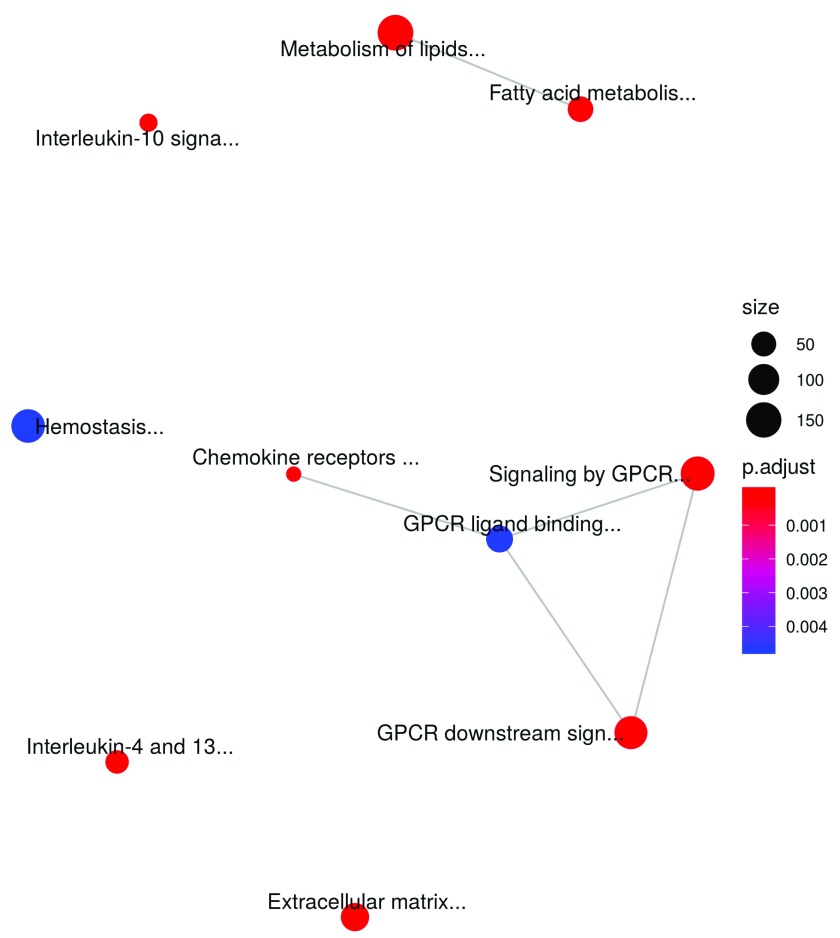
Enriched Reactome pathways enrichment results as a graph.

Again, the graph in Figure 18 shows pathways related to signalling and immune response.

The package *clusterProfiler* ^27^ can also perform these analyses using downloaded KEGG data. Furthermore, the package *EnrichmentBrowser* ^28^ additionally offers network-based enrichment analysis of individual pathways. This allows the mapping of the expression data at hand to known regulatory interactions.

## Session information

As the last part of this document, we call the function *sessionInfo*, which reports the version numbers of R and all the packages used in this session. It is good practice to always keep such a record of this as it will help to track down what has happened in case an R script ceases to work or gives different results because the functions have been changed in a newer version of one of your packages. By including it at the bottom of a script, your reports will become more reproducible.

The session information should also *always* be included in any emails to the Bioconductor support site along with all code used in the analysis.

gc()

               used   (Mb) gc trigger (Mb)  max used (Mb)
   Ncells  11237194  600.2  2.051e+07 1095 2.051e+07 1095
   Vcells 425006866 3242.6  1.243e+09 9486 1.243e+09 9486

length(getLoadedDLLs())

   [1] 98

sessionInfo()

   R version 3.5.0 (2018-04-23)
   Platform: x86_64-pc-linux-gnu (64-bit)
   Running under: Ubuntu 16.04.4 LTS

   Matrix products: default
   BLAS: /usr/lib/openblas-base/libblas.so.3
   LAPACK: /usr/lib/libopenblasp-r0.2.18.so

   locale:
    [1] LC_CTYPE=en_US.UTF-8       LC_NUMERIC=C
    [3] LC_TIME=de_DE.UTF-8        LC_COLLATE=en_US.UTF-8
    [5] LC_MONETARY=de_DE.UTF-8    LC_MESSAGES=en_US.UTF-8
    [7] LC_PAPER=de_DE.UTF-8       LC_NAME=C
    [9] LC_ADDRESS=C               LC_TELEPHONE=C
   [11] LC_MEASUREMENT=de_DE.UTF-8 LC_IDENTIFICATION=C

   attached base packages:
    [1] grid      stats4    parallel  stats     graphics  grDevices utils
    [8] datasets  methods   base

   other attached packages:
    [1] Rgraphviz_2.24.0                     bindrcpp_0.2.2
    [3] hexbin_1.27.2                        openxlsx_4.1.0
    [5] genefilter_1.62.0                    matrixStats_0.53.1
    [7] stringr_1.3.1                        tidyr_0.8.1
    [9] dplyr_0.7.5                          pheatmap_1.0.10
   [11] RColorBrewer_1.1-2                   geneplotter_1.58.0
   [13] annotate_1.58.0                      XML_3.98-1.11
   [15] lattice_0.20-35                      ggplot2_2.2.1.9000
   [17] gplots_3.0.1                         clusterProfiler_3.8.1
   [19] ReactomePA_1.24.0                    topGO_2.32.0
   [21] SparseM_1.77                         GO.db_3.6.0
   [23] graph_1.58.0                         limma_3.36.1
   [25] arrayQualityMetrics_3.36.0           hugene10sttranscriptcluster.db_8.7.0
   [27] org.Hs.eg.db_3.6.0                   AnnotationDbi_1.42.1
   [29] pd.hugene.1.0.st.v1_3.14.1           DBI_1.0.0
   [31] oligo_1.44.0                         RSQLite_2.1.1
   [33] Biostrings_2.48.0                    XVector_0.20.0
   [35] IRanges_2.14.10                      S4Vectors_0.18.2
   [37] ArrayExpress_1.40.0                  oligoClasses_1.42.0
   [39] Biobase_2.40.0                       BiocGenerics_0.26.0
   [41] maEndToEnd_0.99.0                    knitr_1.20
   [43] BiocStyle_2.8.2

   loaded via a namespace (and not attached):
     [1] utf8_1.1.4                  tidyselect_0.2.4
     [3] htmlwidgets_1.2             beadarray_2.30.0
     [5] BiocParallel_1.14.1         devtools_1.13.5
     [7] munsell_0.4.3               codetools_0.2-15
     [9] preprocessCore_1.42.0       units_0.5-1
    [11] withr_2.1.2                 colorspace_1.3-2
    [13] GOSemSim_2.6.0              BiocInstaller_1.30.0
    [15] highr_0.6                   rstudioapi_0.7
    [17] setRNG_2013.9-1             DOSE_3.7.0
    [19] labeling_0.3                git2r_0.21.0
    [21] GenomeInfoDbData_1.1.0      hwriter_1.3.2
    [23] bit64_0.9-7                 rprojroot_1.3-2
    [25] xfun_0.1                    affxparser_1.52.0
    [27] R6_2.2.2                    GenomeInfoDb_1.16.0
    [29] illuminaio_0.22.0           gridSVG_1.6-0
    [31] bitops_1.0-6                fgsea_1.6.0
    [33] DelayedArray_0.6.0          assertthat_0.2.0
    [35] scales_0.5.0                ggraph_1.0.1
    [37] nnet_7.3-12                 enrichplot_1.1.0
    [39] gtable_0.2.0                Cairo_1.5-9
    [41] affy_1.58.0                 rlang_0.2.1
    [43] splines_3.5.0               lazyeval_0.2.1
    [45] acepack_1.4.1               checkmate_1.8.5
    [47] yaml_2.1.19                 reshape2_1.4.3
    [49] backports_1.1.2             qvalue_2.12.0
    [51] Hmisc_4.1-1                 tools_3.5.0
    [53] bookdown_0.7                affyio_1.50.0
    [55] ff_2.2-14                   ggridges_0.5.0
    [57] Rcpp_0.12.17                plyr_1.8.4
    [59] base64enc_0.1-3             zlibbioc_1.26.0
    [61] purrr_0.2.5                 RCurl_1.95-4.10
    [63] rpart_4.1-13                openssl_1.0.1
    [65] viridis_0.5.1               cowplot_0.9.2
    [67] SummarizedExperiment_1.10.1 ggrepel_0.8.0
    [69] cluster_2.0.7-1             tinytex_0.5
    [71] magrittr_1.5                data.table_1.11.4
    [73] DO.db_2.9                   reactome.db_1.64.0
    [75] evaluate_0.10.1             xtable_1.8-2
    [77] gcrma_2.52.0                gridExtra_2.3
    [79] compiler_3.5.0              tibble_1.4.2
    [81] crayon_1.3.4                KernSmooth_2.23-15
    [83] htmltools_0.3.6             Formula_1.2-3
    [85] BiocWorkflowTools_1.6.1     udunits2_0.13
    [87] tweenr_0.1.5                MASS_7.3-50
    [89] rappdirs_0.3.1              Matrix_1.2-14
    [91] cli_1.0.0                   vsn_3.48.1
    [93] gdata_2.18.0                bindr_0.1.1
    [95] igraph_1.2.1                GenomicRanges_1.32.3
    [97] pkgconfig_2.0.1             rvcheck_0.1.0
    [99] foreign_0.8-70              foreach_1.4.4
   [101] BeadDataPackR_1.32.0        affyPLM_1.56.0
   [103] digest_0.6.15               rmarkdown_1.9
   [105] base64_2.0                  fastmatch_1.1-0
   [107] htmlTable_1.12              gtools_3.5.0
   [109] graphite_1.26.1             jsonlite_1.5
   [111] viridisLite_0.3.0           pillar_1.2.3
   [113] httr_1.3.1                  survival_2.42-3
   [115] glue_1.2.0                  zip_1.0.0
   [117] UpSetR_1.3.3                iterators_1.0.9
   [119] bit_1.1-14                  ggforce_0.1.2
   [121] stringi_1.2.2               blob_1.1.1
   [123] latticeExtra_0.6-28         caTools_1.17.1
   [125] memoise_1.1.0

R markdown document to reproduce the results obtained in the article. This file allows the reader to reproduce the analysis results obtained in the article. Click here for additional data file. Copyright: © 2018 Klaus B and Reisenauer S 2018 Data associated with the article are available under the terms of the Creative Commons Zero "No rights reserved" data waiver (CC0 1.0 Public domain dedication).

## Data and software availability

This article is based on an R markdown file (MA-Workflow.Rmd) which is available as Dataset 1 (Dataset 1. R markdown document to reproduce the results obtained in the article, 10.5256/f1000research.8967.d208076) ^29^ and is currently available via the development version (3.8) of Bioconductor. This will become the release version in October 2018. The .Rmd file allows the reader to reproduce the analysis results obtained in this article. All data analyzed are downloaded from ArrayExpress.
