# Supplementary material for: An end to end workflow for differential gene expression using Affymetrix microarrays
Source: F1000Res. 2018 Jul 3;5:1384. Originally published 2016 Jun 15. [Version 2] doi: 10.12688/f1000research.8967.2 (PMC6063319; doi:10.12688/f1000research.8967.2)
Supplement: Peer review history

# Peer review: Referee response for version 2

**DOI:** 10.5256/f1000research.16727.r35714

**Published:** 2018-07-25

**Authors:** Andrea Rau

**Affiliations:** 1 UMR 1313 GABI (Animal Genetics and Integrative Biology), French National Institute for Agricultural Research (INRA), Jouy-en-Josas, France

The authors have done a nice job of responding to the reviewers' comments, and this second version of the manuscript is greatly improved.

I have read this submission. I believe that I have an appropriate level of expertise to confirm that it is of an acceptable scientific standard.

# Peer review: Referee response for version 2

**DOI:** 10.5256/f1000research.16727.r35715

**Published:** 2018-07-11

**Authors:** James W. MacDonald

**Affiliations:** 1 Department of Environmental and Occupational Health Sciences, University of Washington, Seattle, WA, USA

The second version of this article is much improved from the first. The explanations are simplified, and much of what I considered to be extraneous has been removed. I only have a few minor points that could be addressed.

 It's not necessary (ever) to use install.packages() or the remotes package directly, for any Bioconductor package or really any package. Installing the maEndToEnd package can be accomplished by simply using

 biocLite("b-klaus/maEndToEnd")

 There is some complexity here, as you may need to install some of the dependencies for the maEndToEnd package, but that is a consequence of getting the package from GitHub rather than a failing of biocLite(). End users will have to install the dependencies regardless, so something like

 deps  <- strsplit(packageDescription("maEndToEnd")$Depends,",\\n")[[1]][-1]

 deps <- deps[!deps %in% row.names(installed.packages())]

 biocLite(deps)

 would do the trick.

 Making the RLE plot could be simplified by using oligo::basicPLM rather than oligo::rma

 RLE(basicPLM(raw_data, normalize = FALSE))

 While it is true that RMA summarization for Exon ST arrays can be carried out at the 'full' or 'extended' level, that is not true for Gene ST arrays, which can only be summarized at the 'core' and 'probeset' level, so I would remove that from the section titled 'RMA calibration of the data' .

 Under the linear models section, the resource you call 'genomics class' is actually the Biomedical Data Science course and is authored by Mike Love and Rafael Irizarry (not Michael Irizzary).

I have read this submission. I believe that I have an appropriate level of expertise to confirm that it is of an acceptable scientific standard.

# Peer review: Referee response for version 1

**DOI:** 10.5256/f1000research.9647.r14390

**Published:** 2016-07-08

**Authors:** Andrea Rau

**Affiliations:** 1 UMR 1313 GABI (Animal Genetics and Integrative Biology), French National Institute for Agricultural Research (INRA), Jouy-en-Josas, France

Klaus illustrates an analysis workflow of Affymetrix microarrays for an experimental design with paired samples from two tissues in two separate diseases. The workflow covers steps including downloading and loading raw CEL files into R/Bioconductor, pre-processing and normalization, differential analysis via limma, and functional/pathway enrichment analysis, with detailed R scripts throughout. The workflow is a nice complement to resources that are already available on similar topics (notably the Limma user's guide) as it unites into a single document topics that have been discussed elsewhere in diverse forums.

Major remarks:

- Although the normalization/linear model/contrast sections are technically correct, I find them to be overly disruptive to the presentation of the analysis. In particular, I would suggest moving the normalization section to an appendix. For the linear models section, presentation of ordinary least squares estimators, matrix notation, distributional assumptions of residuals, etc. seems outside of the scope of this work (or could potentially be presented in an appendix as well). I think that it would be more helpful if, rather than using a separate illustrative example based on the *toycars *data, the author described in detail: 1) the design matrix for the Palmieri example; 2) a linear model for a single gene from the Palmieri data; 3) a linear model fit independently for each gene, sharing information across all genes (limma), with a brief discussion about the advantage of such an approach; 4) a simplified discussion of various contrasts relevant for this particular study.
- I was surprised to see that differential analysis conclusions are based on the raw p-values rather than those adjusted for multiple testing.
- I have two additional suggestions that could be of great practical interest to many readers:

1. I think it would be nice to have a discussion (or at least some references) discussing what can be done if hypotheses are NOT met (e.g., the raw p-values are not uniformly distributed between 0 and 1 for genes under H0). I have in mind the author's recent discussion about using fdrtool to estimate the variance of the null model in the context of differential analyses via DESeq2 (http://www-huber.embl.de/users/klaus/Teaching/DESeq2Predoc2014.html).
2. In addition to the fixed patient effect model presented here, I think it would be interesting to present an alternative strategy possible with *limma *for such a design: estimating correlation within patient blocks using duplicateCorrelation(), see for example section 17.3.6 in the *limma* User's Guide. Note that I am not suggesting a detailed comparison or presentation of mixed models (that is of course well beyond the scope of this work!), but it may be useful to discuss the code/results for such an approach.

Minor remarks:

- I would suggest specifying in the abstract that the study "...compares paired inflamed and non-inflamed colon tissue...", as this was not immediately clear otherwise. In addition, on page 2/paragraph 1, the author writes that the original paper studied "differences between patients suffering UC or CD", which makes it sound like the disease comparison was of interest, when in fact it was the intra-patient tissue differences that were studied. It may in fact be helpful to present the experimental design in greater detail at the start of the paper; it was not clear to me until page 23 that the design actually involved paired samples from individuals.
- Page 12, the author says "In order to infer a cutoff from the data, we inspect the histogram of the median–intensities. We visually fit a central normal distribution given by 0.5 · N(5.1, 1.18) to the probe–wise medians". From the R code, it appears that the distribution fit was N(emp_mu = 5.3, emp_sd = 1.19), where emp_mu and emp_sd were estimated from the median expression values -- this is unclear from the expression "visually fit" in the text description. Also unclear to me is why prop_cental was set to be 0.5?
- The phrase "from the XXXX" is often used when referencing R packages, rather than "from XXXX" or "from the XXX package".
- The comparison to the original paper results (page 26) seems unnecessary.
- I found it a bit distracting that none of the figures are explicitly referred to by number in the text (e.g., "In Figure 1, ...").
- There are occasionally very verbose outputs included in the text (e.g. head(pData(raw_data))) that produce multiple pages of output that are not particularly useful.
- There are a few English typos throughout, e.g. "inflammed" should be "inflamed", "constrains" should be "constraints", "transcriptct clusters" should be "transcript clusters", etc.
- Since a very large number of R packages are loaded for the analysis on page 2, it would likely be helpful to either reduce the number of packages needed to be the strict minimum necessary, or to group packages by theme/functionality via comments, for example:

1. General Bioconductor packages
  library(Biobase)
  library(oligoClasses)
2. Annotation and data packages
  library(pd.hugene.1.0.st.v1)
3. Quality control and pre-processing packages
  library(oligo)
4. Analysis and statistics packages
  library(limma)
  library(mvtnorm)
5. Plotting and color options packageslibrary(gplots)
  library(ggplot2)
6. Formatting/documentation packages
  library(knitr)
  library(BiocStyle)
  library(dplyr)

I have read this submission. I believe that I have an appropriate level of expertise to confirm that it is of an acceptable scientific standard.

# Response

**Published:** 2018-06-14

**Authors:** Bernd Klaus

**Affiliations:** EMBL Heidelberg, Germany

Klaus illustrates an analysis workflow of Affymetrix microarrays for an experimental design with paired samples from two tissues in two separate diseases. The workflow covers steps including downloading and loading raw CEL files into R/Bioconductor, pre-processing and normalization, differential analysis via limma, and functional/pathway enrichment analysis, with detailed R scripts throughout. The workflow is a nice complement to resources that are already available on similar topics (notably the Limma user's guide) as it unites into a single document topics that have been discussed elsewhere in diverse forums.

 #Major remarks:

 # remark 1

     Although the normalization/linear model/contrast sections are technically correct, I find them to be overly disruptive to the presentation of the analysis. In particular, I would suggest moving the normalization section to an appendix. For the linear models section, presentation of ordinary least squares estimators, matrix notation, distributional assumptions of residuals, etc. seems outside of the scope of this work (or could potentially be presented in an appendix as well). I think that it would be more helpful if, rather than using a separate illustrative example based on the toycars data, the author described in detail:

     1) the design matrix for the Palmieri example;

     2) a linear model for a single gene from the Palmieri data;

     3) a linear model fit independently for each gene, sharing information across all genes (limma), with a brief discussion about the advantage of such an approach;

     4) a simplified discussion of various contrasts relevant for this particular study.

 Answer:

 The linear model sections not directly related to the dataset at hand were

 removed from the workflow. In general, we have tried to tailor the revised workflow to

 beginners in R and Bioconductor. Therefore, rather than discussing elaborate

 tools / techniques in detail, we try to point to the relevant literature where

 appropriate.

 This is helpful for the reader who needs to deviate from our workflow

 for his or her own data and maintains a straightforward workflow design.

 Concerning the single points mentioned in the comment above:

 1) The design matrix for the Palmieri example is now explained in detail.

 2) In the subsequent part of the workflow, the advise of the reviewer to implement a linear model for a single gene from the Palmieri data was adopted by fitting a linear model to the "CRAT" gene, and testing it for differential expression.

 3) The advantage of shared information across all genes is discussed briefly when introducing the "eBayes"-method in the "Contrasts and Hypotheses tests" part.

 4) In this workflow, we restrict the contrast analysis to the contrast between non-inflamed and inflamed tissue, as it is the one that is also analyzed in the original paper, and do not include additional contrasts in order to keep the workflow

 concise.

 # remark 2

      

     I was surprised to see that differential analysis conclusions are based on the raw p-values rather than those adjusted for multiple testing.

 Answer: This is indeed a possible cause of confusion. In the workflow, parts of the analysis are based on raw p-values in order to make the results comparable to the original paper. However, we now mention the caveats of this approach more,

 introduce FDRs in a "hands-on" manner

 explicitly and caution against the use of raw p--values in practice.

 For the subsequent enrichment analyses,

 DE genes are identified using an FDR cutoff of 10%.

     I have two additional suggestions that could be of great practical interest to many readers:

 # remark 3

     I think it would be nice to have a discussion (or at least some references) discussing what can be done if hypotheses are NOT met (e.g., the raw p-values are not uniformly distributed between 0 and 1 for genes under H0). I have in mind the author's recent discussion about using fdrtool to estimate the variance of the null model in the context of differential analyses via DESeq2 (http://www-huber.embl.de/users/klaus/Teaching/DESeq2Predoc2014.html).

     

 Answer: As mentioned above, and in concordance with the requests of Jim,

 we tried to avoid introducing any additional (advanced) methods in order

 to not confuse the novice  R/Bioconductor user. Nevertheless,

 for workflow users encountering the problem of unusual p-value distributions,

 we have included references to this phenomenon and its implications by referring to

 the article on false discovery rate estimation by Korbininan Strimmer

 and chapter 1-6 of Efron's book on Large-Scale Inference.

 # remark 4

     In addition to the fixed patient effect model presented here, I think it would be interesting to present an alternative strategy possible with limma for such a design: estimating correlation within patient blocks using duplicateCorrelation(), see for example section 17.3.6 in the limma User's Guide. Note that I am not suggesting a detailed comparison or presentation of mixed models (that is of course well beyond the scope of this work!), but it may be useful to discuss the code/results for such an approach.

 Answer: We acknowledge the advantages in certain applications of a mixed model using duplicateCorrelation() compared to a fixed patient effect model used in this workflow.

 Given that the workflow is tailored towards unexperienced users,

 we consider the fixed patient effect model more intuitively

 understandable. However, we now note explicitly that such an

 analysis could be performed.

 Minor remarks:

 # remark 5

     I would suggest specifying in the abstract that the study "...compares paired inflamed and non-inflamed colon tissue...", as this was not immediately clear otherwise. In addition, on page 2/paragraph 1, the author writes that the original paper studied "differences between patients suffering UC or CD", which makes it sound like the disease comparison was of interest, when in fact it was the intra-patient tissue differences that were studied. It may in fact be helpful to present the experimental design in greater detail at the start of the paper; it was not clear to me until page 23 that the design actually involved paired samples from individuals.

     

 Answer: The experimental design is now described in greater detail in abstract and

 introduction. Also, the sentence that the original paper studied "differences

 between patients suffering from UC or CD" was removed.

    

 # remark 6

     Page 12, the author says "In order to infer a cutoff from the data, we inspect the histogram of the median–intensities. We visually fit a central normal distribution given by 0.5 · N(5.1, 1.18) to the probe–wise medians". From the R code, it appears that the distribution fit was N(emp_mu = 5.3, emp_sd = 1.19), where emp_mu and emp_sd were estimated from the median expression values -- this is unclear from the expression "visually fit" in the text description. Also unclear to me is why prop_cental was set to be 0.5?

 Answer: In an attempt to make low-intensity-filtering more intuitive, we removed

 the fitting a normal distribution the the histogram of the probe-wise medians.

 Instead, we now visually set a vertical cutoff line to the histogram and filter  

 genes with a lower intensity than the cutoff in at least as many samples as the smallest experimental group has.

 # additional minor remarks

      

     The phrase "from the XXXX" is often used when referencing R packages, rather than "from XXXX" or "from the XXX package".

 Answer: Thank you, the respective sentences were corrected accordingly.

      

     The comparison to the original paper results (page 26) seems unnecessary.

 Answer: The comparison to the original paper serves as a "proof of principle"

 for the implemented workflow (to show that what we are doing makes sense).

 Additionally, we wanted to show that it is relatively straightforward

 to re--analyze publicly available microarray data using R and Bioconductor.

      

     I found it a bit distracting that none of the figures are explicitly referred to by number in the text (e.g., "In Figure 1, ...").

     

   Answer: Thank you, the respective parts

   were changed accordingly.   

      

     There are occasionally very verbose outputs included in the text (e.g. head(pData(raw_data))) that produce multiple pages of output that are not particularly useful.

     

 Answer: The output was shortened where appropriate. The pdata call in

 particular was retained, as we feel that it is interesting to the readers

 to see what kind of information comes with a dataset from Array Express.

      

     There are a few English typos throughout, e.g. "inflammed" should be "inflamed", "constrains" should be "constraints", "transcriptct clusters" should be "transcript clusters", etc.

     

 Answer: Thanks, we tried to perform thorough spell-checking in the revised version.

      

     Since a very large number of R packages are loaded for the analysis on page 2, it would likely be helpful to either reduce the number of packages needed to be the strict minimum necessary, or to group packages by theme/functionality via comments, for example:

     General Bioconductor packages

     library(Biobase)

     library(oligoClasses)

      

     Annotation and data packages

     library(pd.hugene.1.0.st.v1)

      

     Quality control and pre-processing packages

     library(oligo)

      

     Analysis and statistics packages

     library(limma)

     library(mvtnorm)

      

     Plotting and color options packageslibrary(gplots)

     library(ggplot2)

      

     Formatting/documentation packages

     library(knitr)

     library(BiocStyle)

     library(dplyr)

 Answer: Thank you for this very useful suggestion, we implemented it in the revised

 version; the package-import statements at the beginning of the workflow is now

 grouped by topic.

# Peer review: Referee response for version 1

**DOI:** 10.5256/f1000research.9647.r14392

**Published:** 2016-07-05

**Authors:** James W. MacDonald

**Affiliations:** 1 Department of Environmental and Occupational Health Sciences, University of Washington, Seattle, WA, USA

This manuscript is intended to take the reader through a complete analysis of Affymetrix Gene ST arrays, based on a set of arrays downloaded from ArrayExpress. The author covers each step from quality control of the raw data all the way to making comparisons using linear models and testing and visualizing pathways or gene sets.

**Major comments**

 While this manuscript is technically correct (e.g., the code does what the author claims, the explanatory text is valid), I am not sure it is as useful as it could be for its intended audience. In other words, this manuscript is intended to provide an inexperienced reader (inexperienced in either R/Bioconductor or statistics or both) with a road map they can follow to learn how to analyze microarray data. However, both the code and the statistical explanations are far too complex to be useful for such a person.

 As an example, the author explains in mathematical terms what the background correction and summarization steps are intended to accomplish. While this is an important step in the analysis, this could instead be explained in heuristic terms that would be far more approachable for a less statistically savvy audience, while still conveying the general idea.

 The section on linear modeling and design matrices are similarly impenetrable for non-statisticians. The *limma User's Guide* has dozens of examples of model matrices, with clear interpretations of the model coefficients. Yet as the author notes, this is probably the number one question on the Bioconductor support site. Rather than re-explaining something that most people clearly don't understand, it would be much more helpful to focus on a single model matrix, and provide a clear, heuristic explanation. The easiest to understand is the most basic orthogonal model that computes the mean of each group, followed by a contrast matrix to make comparisons of interest. 

 The overview on linear models is well beyond the scope of this manuscript, and should be excised. The same is true for the section on testing general linear hypotheses. Ideally, an analyst using these tools would understand what they are doing from a statistical perspective, but it's difficult enough for a novice user to comprehend what the code is doing without trying to also understand the statistics.

 Similarly, the code is more complex than necessary. If the goal is to teach novice users about Bioconductor packages, then the code should be restricted as much as possible to those packages and base R. While ggplot2 style graphics, magrittr style function piping and dplyr two-table verbs may be useful for more advanced R users, in this context they are an added distraction. 

 An example of overly-complex code is the filtering step. There are any number of ways to filter out genes that are arguably not expressed. The example is a very sophisticated way to perform this task, but a novice who is just learning doesn't require sophistication, they require something they can understand. Choosing a cutoff based on the distribution of probesets across each array, and then filtering all genes where fewer than 14 samples exceed this cutoff is not very sophisticated, but it is easy to understand, and would only require a few lines of code.

 The learning curve for R and Bioconductor is steep, and making this manuscript both simpler in terms of the code, and more focused by excluding most of the statistics and any example analyses that do not involve the microarray data would make it more approachable for inexperienced users.

      

**Minor comments**

 In the download step, getAE() is used to download the data, but then the SDRF file is downloaded directly. This is confusing, as getAE() has already downloaded that file. Is there a particular reason for the extra step?

 To test for model matrices that are not full rank, it's easier to use either nonEstimable() or is.fullrank() from the limma package.

**Conclusion**

 As noted above, this manuscript is technically accurate, and the code does exactly what the author claims. But it is too ambitious for the intended audience. Most people who could benefit from this manuscript are not statistically savvy, and the statistical sections will simply confuse them. In addition, it is not likely that many potential readers will be familiar with packages from the 'Hadleyverse', and incorporating those packages in the manuscript makes the code more difficult to understand.

 Paring the manuscript down to very simple heuristic statistical explanations, and limiting the code to functions from base R and the various Bioconductor packages being illustrated would make the manuscript more useful for its intended audience.

I have read this submission. I believe that I have an appropriate level of expertise to confirm that it is of an acceptable scientific standard, however I have significant reservations, as outlined above.

# Response

**Published:** 2018-06-14

**Authors:** Bernd Klaus

**Affiliations:** EMBL Heidelberg, Germany

# **Major comments**

 ## **comment 1**

*While this manuscript is technically correct (e.g., the code does what the author claims, the explanatory text is valid), I am not sure it is as useful as it could be for its intended audience. In other words, this manuscript is intended to provide an inexperienced reader (inexperienced in either R/Bioconductor or statistics or both) with a road map they can follow to learn how to analyze microarray data. However, both the code and the statistical explanations are far too complex to be useful for such a person.*

*As an example, the author explains in mathematical terms what the background correction and summarization steps are intended to accomplish. While this is an important step in the analysis, this could instead be explained in heuristic terms that would be far more approachable for a less statistically savvy audience, while still conveying the general idea.*

 Answer:  As suggested, the general style of the workflow was changed a lot in order to

 guide inexperienced readers through the analysis of microarray data.

 Code is  explained in greater detail, and all technical parts not directly related

 to the data at hand have either been removed or improved.

 However, we have decided to keep the the mathematical explanation

 of background correction and summarization, as we feel that the (rather simple)

 formulas are easier to understand than text.

 ## **comment 2**

*The section on linear modeling and design matrices are similarly impenetrable for non-statisticians. The limma User's Guide has dozens of examples of model matrices, with clear interpretations of the model coefficients. Yet as the author notes, this is probably the number one question on the Bioconductor support site. Rather than re-explaining something that most people clearly don't understand, it would be much more helpful to focus on a single model matrix, and provide a clear, heuristic explanation. The easiest to understand is the most basic orthogonal model that computes the mean of each group, followed by a contrast matrix to make comparisons of interest.*

**

*The overview on linear models is well beyond the scope of this manuscript, and should be excised. The same is true for the section on testing general linear hypotheses. Ideally, an analyst using these tools would understand what they are doing from a statistical perspective, but it's difficult enough for a novice user to comprehend what the code is doing without trying to also understand the statistics.*

 Answer: We gladly adopted this point of criticism and eliminated

 the rather technical part in which the theory behind design matrices

 and linear modelling is explained. Instead, we focused on explaining

 the design matrix we haved used in the dataset at hand in easy-to-follow terms

 and present its use in the context of single linear model for a single gene.

 ## **comment 3**

*Similarly, the code is more complex than necessary. If the goal is to teach novice users about Bioconductor packages, then the code should be restricted as much as possible to those packages and base R. While ggplot2 style graphics, magrittr style function piping and dplyr two-table verbs may be useful for more advanced R users, in this context they are an added distraction.*

**

*An example of overly-complex code is the filtering step. There are any number of ways to filter out genes that are arguably not expressed. The example is a very sophisticated way to perform this task, but a novice who is just learning doesn't require sophistication, they require something they can understand. Choosing a cutoff based on the distribution of probesets across each array, and then filtering all genes where fewer than 14 samples exceed this cutoff is not very sophisticated, but it is easy to understand, and would only require a few lines of code.*

 Answer: While not all of the code from the packages like ggplot2, magrittr and dplyr

 was removed, an effort was made to explain the code in more detail

 in order to give the inexperienced user the possibility to understand what it does. In

 particular, we think that ggplot2 is by now very commonly used and probably becoming a defacto standard plotting package.

 In the low-intensity filtering step, the fitting of a normal distribution

 to the histogram was eliminated for the sake of clarity. Instead,

 we now visually set a vertical cutoff line to the histogram and filter

 genes with a lower intensity than the cutoff in at least as many samples

 as the smallest experimental group has.

 ## **comment 4**

*The learning curve for R and Bioconductor is steep, and making this manuscript both simpler in terms of the code, and more focused by excluding most of the statistics and any example analyses that do not involve the microarray data would make it more approachable for inexperienced users.*

 Answer: Thank you for this useful comment. We have made some effort to tailor

 the revised  version to the beginner in R / Bioconductor and / or statistics.

 The revised article contains a more focused workflow with more in depth

 explanations of the code.

 # **Minor comments**

*In the download step, getAE() is used to download the data, but then the SDRF file is downloaded directly. This is confusing, as getAE() has already downloaded that file. Is there a particular reason for the extra step?*

 Answer: There is no particular reason for this step, the code has been changed

 in order to import the SDRF file from the already downloaded data.

*To test for model matrices that are not full rank, it's easier to use either nonEstimable() or is.fullrank() from the limma package.*

 Answer: This section was removed, as it is quite technical and

 is really more of a "sanity check" than a necessity.
